# SOX9 is an atypical intestinal tumor suppressor controlling the oncogenic Wnt/ß-catenin signaling

**DOI:** 10.18632/oncotarget.10573

**Published:** 2016-07-13

**Authors:** Corinne Prévostel, Cyrine Rammah-Bouazza, Hélène Trauchessec, Lucile Canterel-Thouennon, Muriel Busson, Marc Ychou, Philippe Blache

**Affiliations:** ^1^ IRCM, Institut de Recherche en Cancérologie de Montpellier, Montpellier, France; ^2^ INSERM, U1194, Montpellier, France; ^3^ Université de Montpellier, Montpellier, France; ^4^ Institut régional du Cancer de Montpellier, Montpellier, France; ^5^ Université de Montpellier, UMR 5237, Centre de Recherche de Biochimie Macromoléculaire, CNRS, Montpellier, France; ^6^ Centre Hospitalier Régional Universitaire (CHU) de Montpellier, Montpellier, France; ^7^ Institut Régional du Cancer de Montpellier (ICM)-Val d'Aurelle, Montpellier, France

**Keywords:** SOX9, colorectal cancer, tumor suppressor, Wnt/ß-catenin inhibition, c-myc inhibition

## Abstract

SOX9 inactivation is frequent in colorectal cancer (CRC) due to *SOX9* gene mutations and/or to ectopic expression of MiniSOX9, a dominant negative inhibitor of SOX9. In the present study, we report a heterozygous L142P inactivating mutation of *SOX9* in the DLD-1 CRC cell line and we demonstrate that the conditional expression of a wild type SOX9 in this cell line inhibits cell growth, clonal capacity and colonosphere formation while decreasing both the activity of the oncogenic Wnt/ß-catenin signaling pathway and the expression of the c-myc oncogene. This activity does not require SOX9 transcriptional function but, rather, involves an interaction of SOX9 with nuclear ß-catenin. Furthermore, we report that SOX9 inhibits tumor development when conditionally expressed in CRC cells injected either subcutaneous or intraperitoneous in BALB/c mice as an abdominal metastasis model. These observations argue in favor of a tumor suppressor activity for SOX9. As an siRNA targeting SOX9 paradoxically also inhibits DLD-1 and HCT116 CRC cell growth, we conclude that there is a critical level of endogenous active SOX9 needed to maintain CRC cell growth.

## INTRODUCTION

SOX9 is the most extensively studied SOX transcription factor in the intestinal epithelium where it is expressed at the base of the crypts *i.e.* in the stem/progenitor compartment of both the small intestine and the colon [1–4]. SOX9 has a critical role in the control of intestinal epithelial cell proliferation as shown by hyperplasia and dysplasia observed in response to the *Sox9* gene knock out targeted in the intestinal epithelium [2]. This observation is paradoxical given the fact that SOX9 is present in CRC [5, 6]. Nevertheless, it has to be taken into account that SOX9 transcriptional activity is low in intestinal tumor cell lines [7] and this might be due, at least partly, to inactivating mutations of the SOX9 gene [8], but also to our discovery of MiniSOX9 in colon cancer cells. MiniSOX9 is indeed a SOX9 splice variant that behaves as a dominant negative with respect to SOX9 while competing with SOX9 for DNA binding [9]. Thus, both SOX9 mutations and MiniSOX9 expression are likely to contribute to SOX9 inactivation in CRC.

SOX9 was identified as a downstream target, but also as an inhibitor of the oncogenic Wnt/ß-catenin pathway in intestinal epithelial cells [1]. The Wnt/ß-catenin signaling is a constitutively activated pathway in the inherited colorectal cancer (FAP) and in up to 80% of sporadic colorectal cancers (CRC) due to inactivating mutations of the adenomatous polyposis coli (APC) tumor suppressor gene. APC is a component of the ß-catenin degradation complex whose mutations are indeed now clearly recognized as early and sufficient events to promote intestinal tumor development [10]. The precise mechanism whereby SOX9 suppresses the activity of the Wnt/ß-catenin signaling is still not completely solved, but few and sometimes conflicting studies suggest the involvement of several mechanisms including gene expression, protein-protein interactions and the regulation of protein stability. As a transcription factor, SOX9 is primarily expected to directly activate the expression of target genes potentially able to impact on the activity of the Wnt/ß-catenin pathway. For example, CEACAM1 clearly exhibits a suppressive activity on the Wnt/ß-catenin signaling [11] and is a direct target gene of SOX9 in the intestinal epithelium [12]. More recently, two independent studies [13, 14] reported a direct interaction between SOX9 and ß-catenin resulting in the inhibition of ß-catenin transcriptional activity, but there is still controversy as to whether or not this inhibition is due to ß-catenin degradation by the proteasome machinery. Besides, it is still not clear whether SOX9 anti-tumor activity in the intestine is mainly due to its conventional transcription factor activity or to its ability to directly inhibit the Wnt/ß-catenin signaling pathway. In the present study, we show, both *in vitro* and *in vivo,* SOX9 anti-tumor suppressor activities in CRC cells and we demonstrate that SOX9 binds physically with ß-catenin, inhibits the activity of the oncogenic Wnt/ß-catenin signaling pathway by removing ß-catenin from the chromatin and decreases expression of the c-myc oncogene, the prime target gene of the Wnt/ß-catenin pathway.

## RESULTS

### Inactivating mutations of SOX9 in CRC and CRC cell lines, including DLD-1

**Figure 1:**
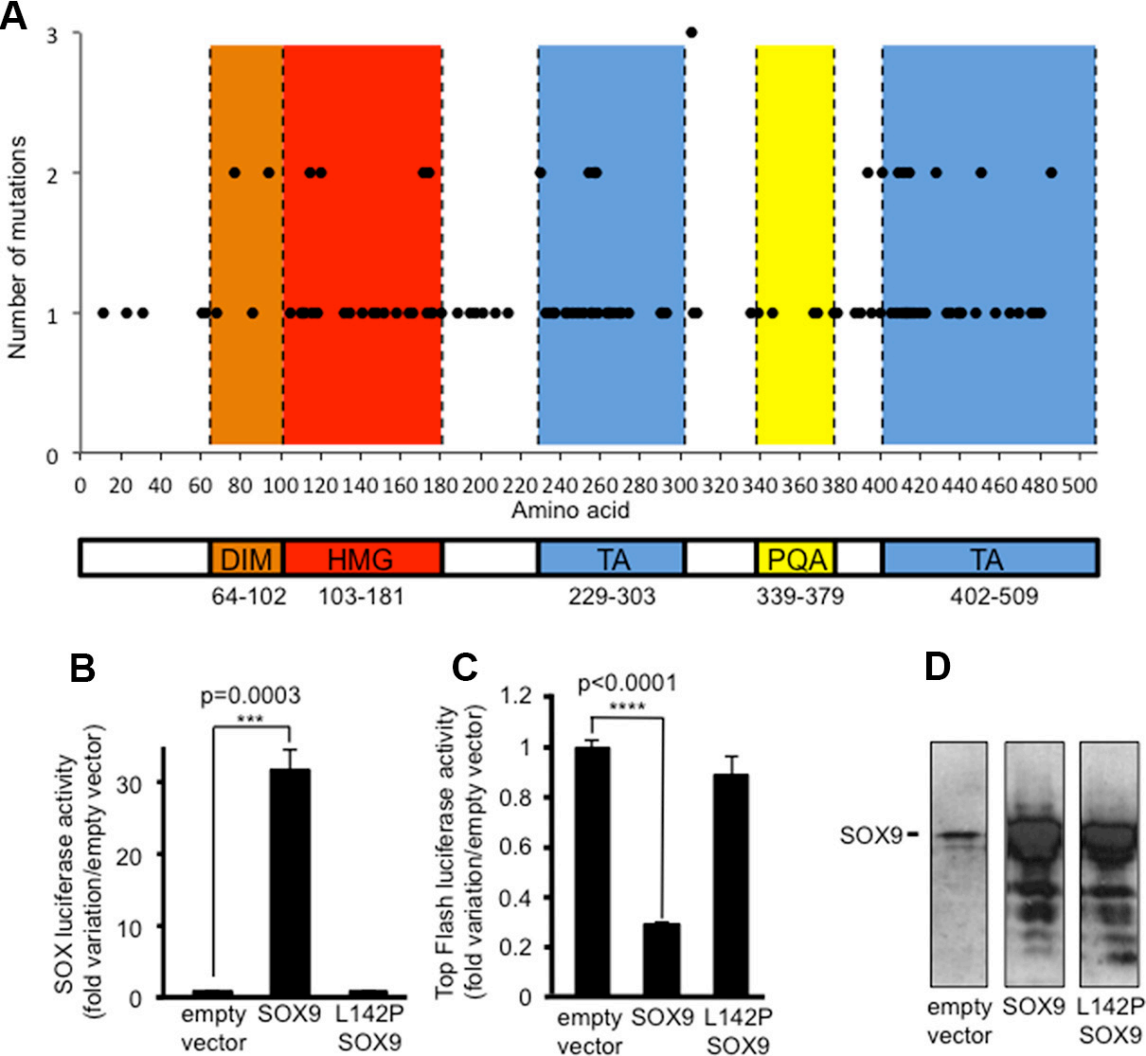
Inactivating mutations of SOX9 in CRC cells including DLD-1 (**A**) SOX9 mutations census according to *http://cancer.sanger.ac.uk/cosmic/*. Note that none of the functional domains are spared by mutations (DIM: dimerization domain (orange); HMG: high mobility group domain/DNA-binding (red), TA: transactivation domains (blue); PQA: proline, glutamine and alanine-rich domain (yellow). (**B**) SOX-luciferase reporter assay (*n* = 3) showing the loss of SOX9 transcriptional activity due to the L142P mutation in DLD-1 cells. (**C**) Top Flash luciferase reporter assay (*n* = 3) showing the loss of SOX9 induced inhibition of the Wnt/ß-catenin signaling pathway activity due to the L142P mutation in DLD-1 cells. (**D**)Visualization by western blot of the SOX9 and L142P-SOX9 transfection efficiency by using the anti-Cter-SOX9 antibody.

It was recently reported that inactivating mutations of *SOX9* are frequent in CRC [8], a situation observed for 25 among 216 human CRCs analyzed in the COAD-US project (11.57%) (http://dcc.icgc.org/web/). *SOX9* mutations are also frequent in colorectal cancer cell lines (14/70) [15] and according to the authors, the heterogeneity of these mutations reveals an anticipated tumor suppressor signature for SOX9 as for APC, TP53 and SMAD4. The heterogeneity of SOX9 mutations in primary colorectal cancers (http://cancer.sanger.ac.uk/cosmic/) is illustrated in Figure 1A and clearly indicates firstly, that none of the SOX9 domains are spared by mutations and secondly, that the most impacted domains are the DNA binding domain (HMG) and the trans-activating domains (TA). The potential impact of those mutations on SOX9 activity is reported in Table S1.

We previously described a mutation that affects the splicing donor site of intron2 of *SOX9* in the DLD-1 cell line [9]. In the present study, we report an additional inactivating mutation on the same allele while the second allele of *SOX9* remains unaffected. This mutation is a leucine to proline substitution (L142P) located in position 142 of SOX9 within the DNA binding domain. It is responsible for a weak transcriptional activity of SOX9 as evidenced by the weak SOX-dependent luciferase activity of the transiently expressed L142P mutant compared with the wild type SOX9 (Figure 1B). Thus, it is likely that the L142P inactivating mutation of SOX9 and the expression of MiniSOX9 [9] both contribute not only to a weak SOX9 transcriptional activity in DLD-1 cells (Figure 1B, empty vector lane), but also to a loss of the SOX9 dependent inhibition of the activity of the oncogenic Wnt/ß-catenin signaling pathway. Indeed, when expressed at a level similar to a wild type SOX9 (Figure 1D), the L142P mutant is unable to decrease the Top Flash dependent luciferase activity (Figure 1C). Together, these observations imply that DLD-1 is a suitable CRC cell model for providing evidence as to whether or not, restoring SOX9 activity has an anti-oncogenic effect and, if so, how this might impact on the activity of the Wnt/ß-catenin signaling pathway.

### Restoring SOX9 activity changes the DLD-1 tumor cell phenotype

DLD-1 cells were genetically modified in order to express either flagged wild type SOX9 (S9-DLD-1) or MiniSOX9 (MS9-DLD-1) upon doxycycline induction. MiniSOX9 was mainly used as a control as it is devoid of transcriptional activity due to the lack of the SOX9 transactivation domain, but also as it behaves as a dominant negative with respect to SOX9 transcriptional activity [9].

**Figure 2:**
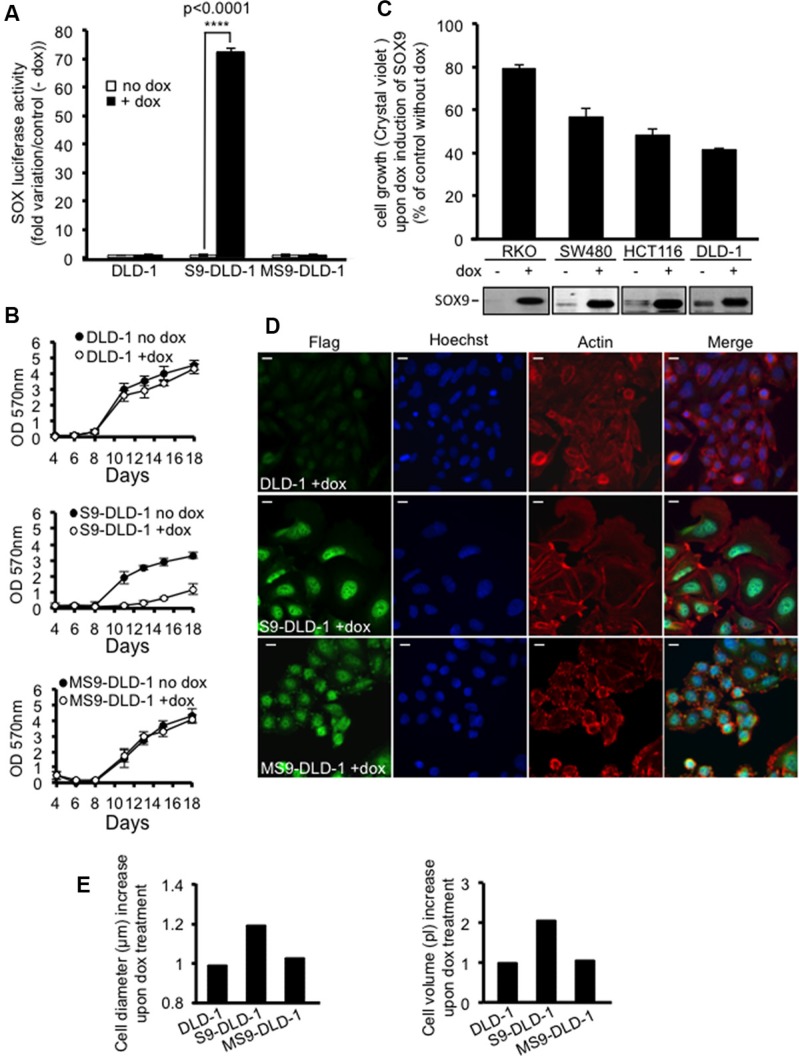
SOX9 inhibits the cell growth and increases the size of DLD-1 cells (**A**) SOX-luciferase assay (*n* = 3) attesting an increase of SOX transcriptional activity in response to doxycycline induced SOX9, but not to MiniSOX9 expression. (**B**) Crystal violet assay (*n* = 12) evidencing a doxycycline-induced decrease of cell growth for S9-DLD-1, but not for either DLD-1 or MS9-DLD-1 cells over 18 days of culture. (**C**) Crystal violet assay (*n* = 6) reporting a SOX9 induced inhibition of cell growth for all the CRC lines tested (RKO, SW480, HCT116 and DLD1); the doxycycline-induced expression of SOX9 is attested by western blotting with the anti-Cter-SOX9 antibody. (**D**) Immunostaining of DLD-1 cells expressing the flag-SOX9 and flag-MS9 constructs upon doxycycline treatment. Note the nuclear localization of SOX9 and MiniSOX9 (flag and Hoechst staining co-localization) and the SOX9 but not MiniSOX9 associated cell size increase (actin staining) (objective X40). Scale bars: 5 μm. (**E**) Representative experiment of normalized scepter data illustrating the increase of cell diameter and volume in response to doxycycline induced SOX9 but not MiniSOX9 expression.

As shown by a flag staining, flagged proteins were both expressed and properly located in DLD-1 cell nuclei after doxycycline treatment (Figure 2D, left panels). Moreover, doxycycline induced SOX9 expression associated with a 70% increase of SOX dependent transcriptional activity (Figure 2A), thus evidencing a functional transcriptional activity for the flagged SOX9. As expected, MiniSOX9 did not have any significant effect on SOX-dependent transcriptional activity (Figure 2A) since it has no SOX9 transcriptional activity and its dominant negative effects are likely to be negligible regarding the weak endogenous activity of SOX9 in DLD-1 cells (Figure 1B). Thus, these genetically modified DLD-1 cells were suitable tools to evaluate the impact of an inducible activity of SOX9 on tumor cell properties.

Firstly, cell growth potential was assessed either by OD readings at 570 nm of solubilized crystal violet stained cells (Figure 2B and 2C), evaluation of cell viability (Figure S1) or counting of cells with a handheld, automated cell counter (Scepter^TM^) (Figure S2). The resulting data clearly demonstrate that SOX9 expression drastically decreased both growth potential (Figures 2B, 2C, S2) and viability of DLD-1 cells (Figure S1). Figures 2C and S1 also further establish that, although less efficient than in DLD-1 cells, this effect was evidenced in all the CRC cell lines tested, whatever their mutational status for SOX9, APC, ß-catenin KRAS or BRAF (Figure S3).

Secondly, both phase contrast observations (Figure S4) and actin staining (Figure 2D, middle panels) were performed on control or doxycycline-induced DLD-1 cells and established that SOX9 expression was associated with a drastic increase in cell size. Scepter measurements (Figures 2E and S5) further confirmed increased volume and diameter of cells in response to the induction of SOX9 expression.

**Figure 3:**
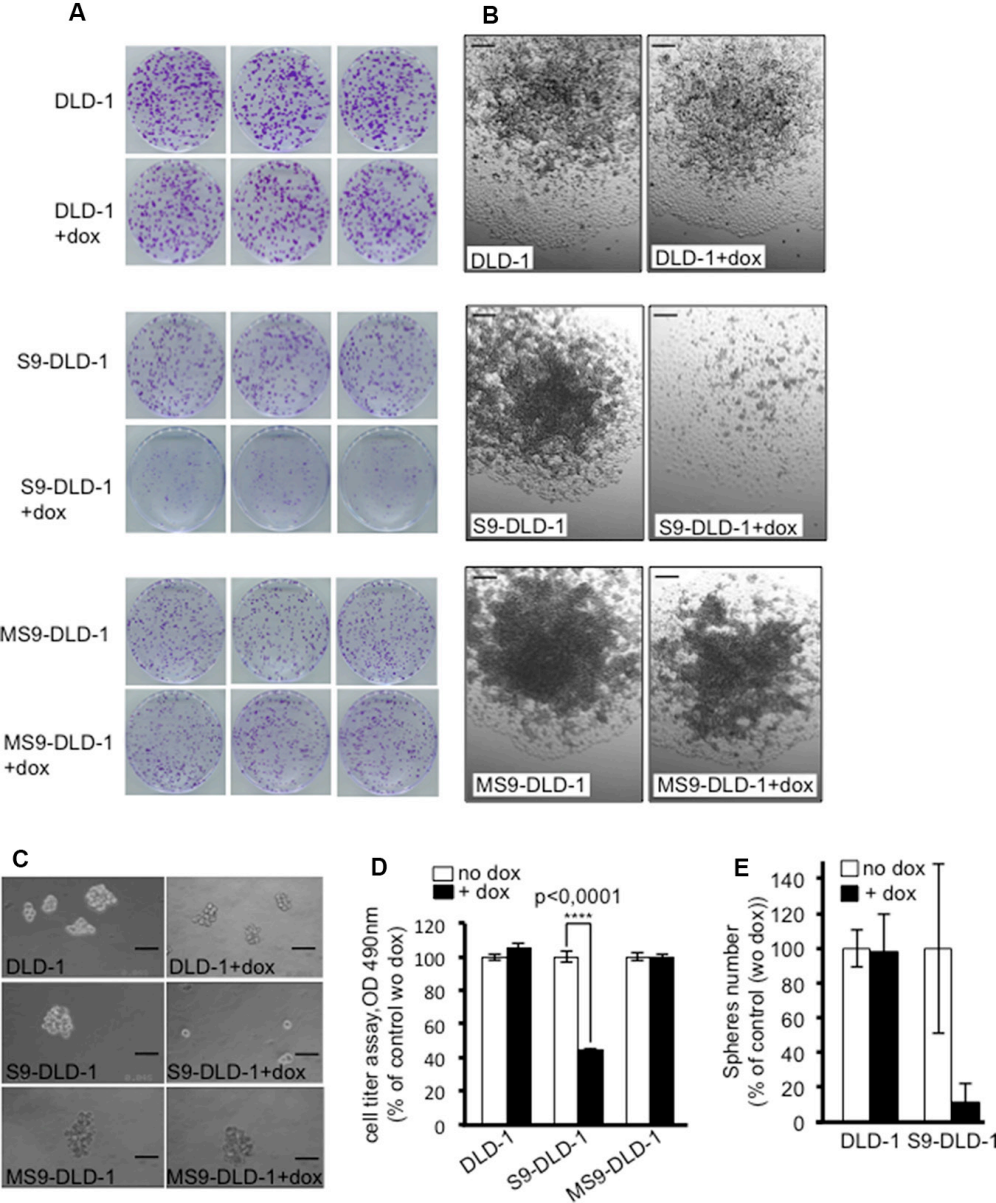
SOX9 decreases the clonal capacity of DLD-1 cells, induces a cell-cell contact inhibition and prevents colonospheres formation (**A**) Crystal violet staining and (**B**) Phase contrast views of DLD-1, S9-DLD-1 and MS9-DLD-1 clones grown for 11 days from 1000 isolated cells in either the presence or absence of doxycycline (objective X10). Scale bars: 100 μm. (**C**) Phase contrast views of DLD-1, S9-DLD-1 and MS9-DLD-1 colonospheres cultured for 11 days in either the absence or presence of doxycycline (objective X10). Scale bars: 100 μm. (**D**) Cell titer assay (*n* = 6) showing the cell viability in DLD-1, S9-DLD-1 and MS9-DLD-1 colonospheres cultured for 11 days in either the absence or presence of doxycycline (**E**) Counting of DLD-1, S9-DLD-1 and MS9-DLD-1 colonospheres grown from 5 starting cells, for 11 days, in 96 well plates in either the absence or presence of doxycycline (*n* = 3).

Thirdly, clonal capacity – *i.e.* the ability of isolated cancer cells cultured in adherent conditions to grow as clones arising from a single cell and proliferating in three dimensions due to a loss of cell-cell contact inhibition- was assessed by counting the number of clones grown from one thousand isolated cells and stained with crystal violet. As shown in Figure 3A, both the number and the size of the clones were decreased upon induction of SOX9 expression, which is consistent with the SOX9-induced inhibition of cell growth potential described above. Phase contrast observations of the clones also further evidenced an inability of DLD-1 cells to grow in the three dimensional directions upon induction of SOX9 expression. Instead, DLD-1 cells seemed only to be capable of growing as monolayers, a common feature of non-transformed epithelial cells (Figure 3B), thus suggesting that SOX9 might be able to restore cell-cell contact inhibition.

It is to be noted that, MiniSOX9 expression had no significant effects on DLD-1 cell growth potential (Figure 2B), cell size (Figures 2D and 2E) or clonal capacity (Figure 3A).

### Restoring SOX9 activity inhibits colonospheres formation

It is now readily admitted that in epithelial cancers, including CRC, self-renewing is driven by a small set of cells termed cancer stem cells (CSC) or tumor initiating cells (TIC). CSC are distinct from the bulk of the cells within the tumor [16] and alone are capable of reinitiating the formation of a tumor, a key condition for cancer recurrence. *In vitro*, this cancer stem potential can be evaluated by assessing the ability of CSC to grow as spheroids (also termed colonospheres for colon CSC) when cultured in limited numbers and under anchorage-independent conditions in a serum-free defined medium supplemented with growth factors [17]. As shown in Figure 3C, (upper left panel) and as previously described for other CRC cell lines, a small set of DLD-1 cells, estimated as about 4% of the whole population were able to grow as colonospheres when cultured for 11 days in such conditions and, also seen in Figure 3C, colonosphere formation was drastically decreased in response to SOX9-inducible expression in DLD-1 cells. In order to further quantify this effect, we set up a cell titer assay reflecting the proportion of living cells grown as colonospheres for 11 days and we could thus estimate that doxycycline induced expression of SOX9 correlated with a 60% decrease of living cells grown as colonospheres (Figure 3D). These results were confirmed by counting colonospheres grown from 5 starting cells in 96 well plates (Figure 3E). Similarly to that observed above for the cell phenotype, doxycycline induced expression of MiniSOX9 had no significant effects on colonosphere formation.

### Restored SOX9 activity inhibits the growth of primary tumors and intraperitoneal metastases

Initially, we performed subcutaneous grafts of S9-DLD-1 cells in nude mice and we observed a significant decrease of tumor growth upon induction of SOX9 expression with doxycycline-supplemented drinking water (Figure S6). Thus, we decided to pursue our study by using syngenic grafts in a mice model with a preserved immune system. Indeed, grafts of the murine CT26 cell line in BALB/c mice are among the most extensively used syngeneic mouse tumor models sharing common features with human CRC. Moreover, the level of SOX9 expression was shown to be very low in CT26 cells in comparison with normal BALB/c mice intestine [18] and DLD-1 cells (Figure S7A), which argued in favor of rather using this model to evaluate the incidence of SOX9 expression on tumor development. As with the DLD-1 cells (see above), CT26 cells were genetically modified in order to express a doxycycline inducible SOX9 (S9-CT26) and also to constitutively express luciferase. A selected S9-CT26 clone expressing moderate SOX9 amounts in response to doxycycline treatment *i.e.* 5.7 fold increase with respect to endogenous SOX9 (Figure S7B) - was tested for SOX9 ability to decrease cell proliferation upon doxycycline induction (Figure S7C).

**Figure 4:**
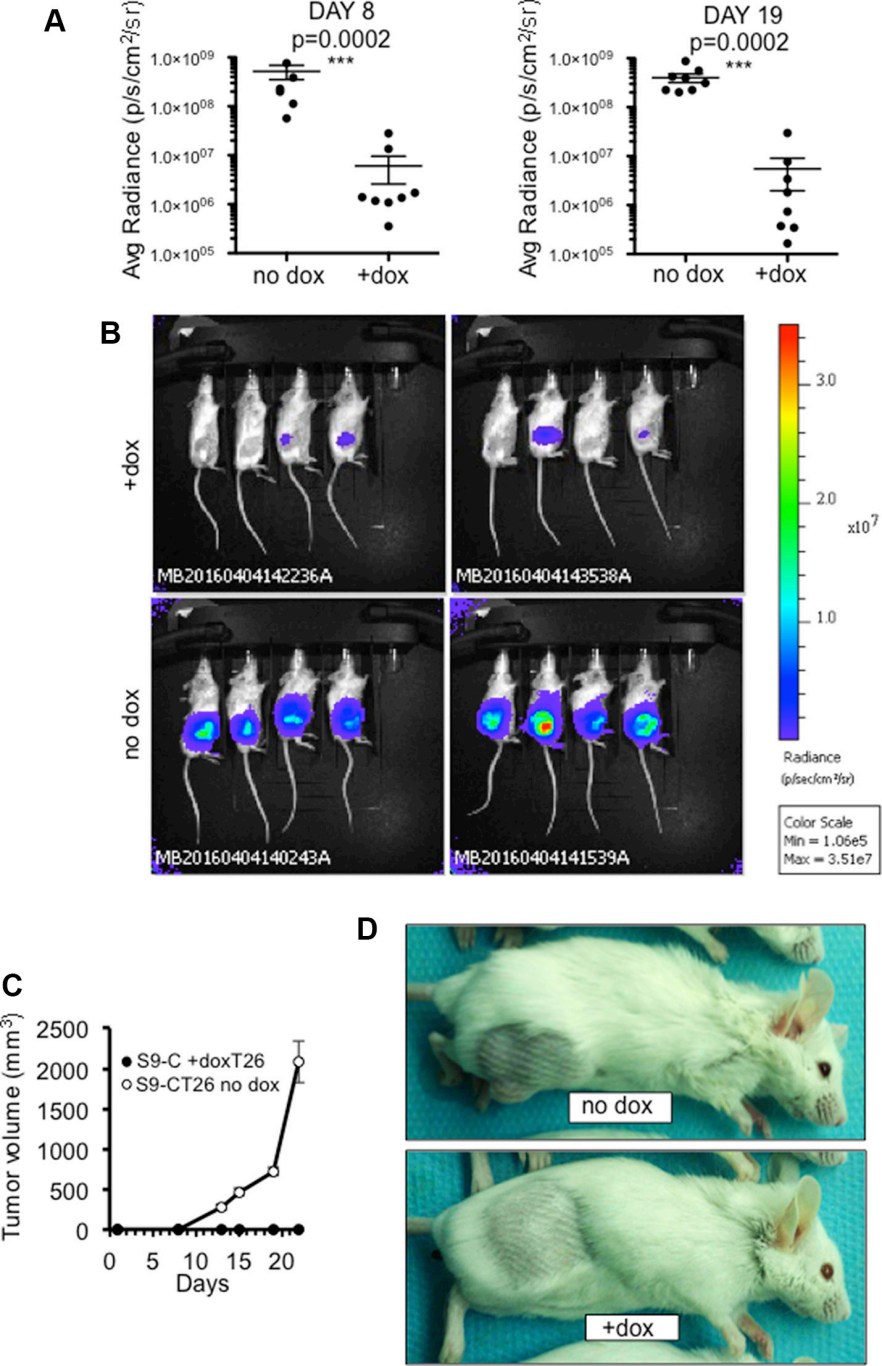
SOX9 inhibits the growth of subcutaneous tumors (**A**) Quantification of the bioluminescence signal (average radiance (p/s/cm2/sr)) associated to the development of subcutaneous tumors 8 and 19 days after transplantation of S9-CT26 cells, upon doxycycline treatment (*n* = 8) or not (*n* = 8). (**B**) Bioluminescence imaging 19 days after subcutaneous transplantation of S9-CT26 cells. (**C**) Measurement of the tumor growth with a caliper at indicated times. (**D**) Representative subcutaneous tumors 22 days after S9-CT26 cells transplantation.

**Figure 5:**
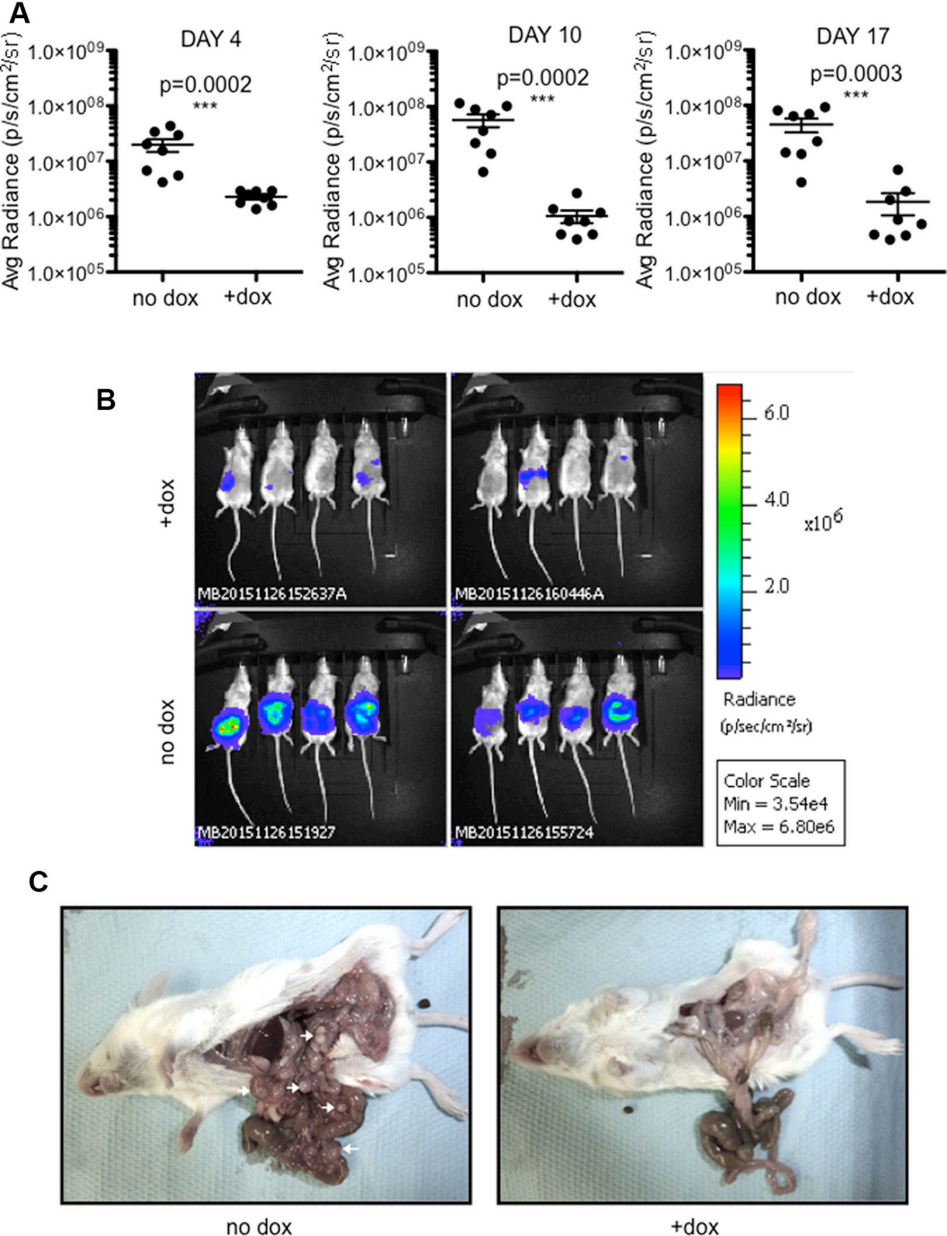
SOX9 inhibits the growth of intraperitoneal metastases (**A**) Quantification of the bioluminescence signal (average radiance (p/s/cm2/sr)) associated to the development of tumors 4, 10, and 17 days after intraperitoneal transplantation of S9-CT26 cells, upon doxycycline treatment (*n* = 8) or not (*n* = 8). (**B**) Bioluminescence imaging 17 days after intraperitoneal transplantation of S9-CT26 cells. (**C**) Representative abdominal tumors (arrows) 17 days after S9-CT26 cells transplantation.

S9-CT26 cells were then grafted subcutaneous in BALB/c mice and, as above, SOX9 expression was induced with doxycycline-supplemented drinking water. Tumor development was followed up in real time by bioluminescence (Figure 4A and 4B) and by measurement of tumor volume (Figure 4C and 4D). As shown in Figure 4A and 4C, both parameters were radically inhibited by SOX9 (*n* = 8). To further investigate the effect of SOX9 on the development of metastasis, S9-CT26 cells were grafted intraperitoneally in BALB/c mice in order to mimic the peritoneal carcinomatosis, a human colorectal cancer metastasis. Quantification of the bioluminescence signals showed variations in the tumor size of control mice (*n* = 8), but clearly evidenced an absence of detectable tumor upon induction of SOX9 expression (*n* = 8) as early as 4 days and up to 17 days post S9-CT26 graft (Figure 5A). Bioluminescence imaging (Figure 5B) and representative examples of the abdominal tumors (Figure 5C) observed in the control mice with respect to doxycycline treated animals (lower panels) illustrate SOX9 ability to inhibit metastasis development. Figure S8 attests that tumor development was not modified by doxycycline treatment of the mice grafted with the CT26 control cells.

### Restored SOX9 activity inhibits the oncogenic Wnt/ß-catenin signaling pathway and decreases the expression of c-myc

**Figure 6:**
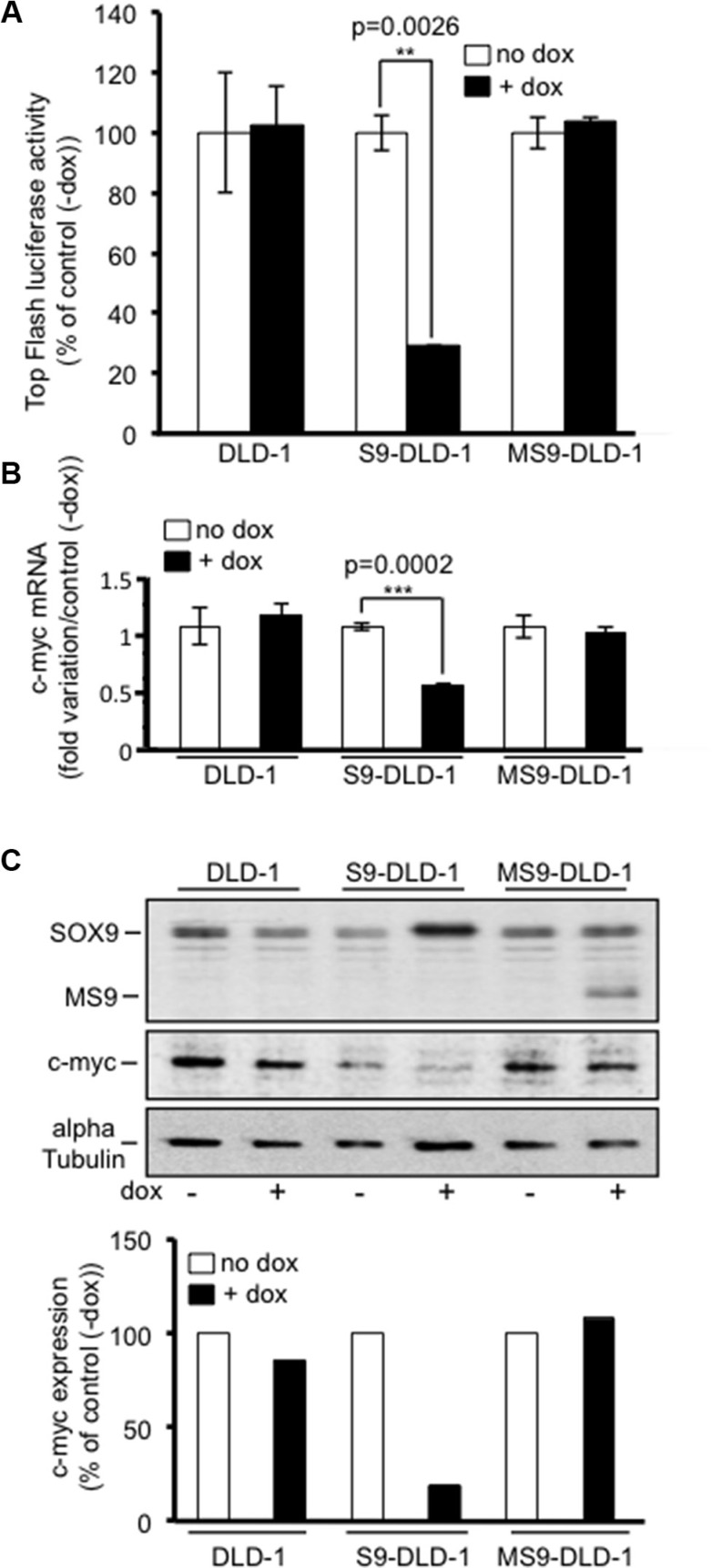
SOX9 decreases the activity of the Wnt/ß-catenin signaling and the expression of c-myc (**A**) Top Flash luciferase reporter assay (*n* = 3) evidencing the decrease of the activity of the Wnt/ß-catenin signaling upon doxycycline induced SOX9, but not MiniSOX9 expression. (**B)** Quantitative RT-PCR analysis reporting c-myc mRNA in response to doxycycline induced SOX9 or MiniSOX9 expression (*n* = 3). (**C)** Western blot analysis of c-myc expression (middle panel) in 2500 cells lysate in response to doxycycline induced SOX9 or MiniSOX9 expression (upper panel) visualized with the anti-NterSOX9/MS9 antibody. Quantification of the c-myc signal normalized to the alpha-tubulin signal by using the ImageJ software (lower panel). Note that SOX9, but not MiniSOX9 expression associates with a decrease of c-myc expression.

The Wnt/ß-catenin signaling pathway was clearly shown to promote the growth potential of both healthy and CRC cells, including CSC, as indicated by the decreased colonosphere formation observed upon ß-catenin targeted siRNA [19, 20]. Since SOX9 inhibits CRC cell growth and colonosphere formation and since SOX9 was previously identified as an inhibitor of the activity of the Wnt/ß-catenin signaling pathway, we suspected that the SOX9 induced inhibition of both cell growth and colonosphere formation might result from an inhibition of the activity of the Wnt/ß-catenin signaling. Consistent with this hypothesis, Figure 6A indicates that the Top flash luciferase activity was more than 70% decreased in S9-DLD-1 cells treated with doxycycline. Doxycycline induced expression of MiniSOX9 did not have any effect on the Top flash luciferase activity. Nevertheless, as reported previously [9], when ß-catenin-Tcf4 activity was stimulated by co-transfecting a stabilized ^33^S ß-catenin, MiniSOX9 increased the ß-catenin-Tcf4 activity (Figure S9).

Among the genes activated by the Wnt/ß-catenin signaling pathway the first one identified was the c-myc proto-oncogene [21] that was later on defined as a master gene for the oncogenic potential of the Wnt/ß-catenin signaling [22]. Regarding the inhibitory effects of SOX9 on the Wnt/ß-catenin signaling pathway in DLD-1 cells, and taking into account that we previously observed an increase of c-myc expression in mice in which the *Sox9* gene was inactivated [2], it was expected that SOX9 might be capable of inhibiting c-myc expression in DLD-1 cells. Consistent with this hypothesis, western blot and quantitative PCR analysis, respectively, presented in Figures 6B and 6C clearly demonstrated a decrease of more than 50% of both the c-myc protein and its encoding mRNA upon 2.5 fold induction of SOX9 level by doxycycline (Figure S10). In contrast, c-myc protein and mRNA expression levels remained unchanged upon MiniSOX9 expression, which is consistent with the fact that MiniSOX9 expression has, in these conditions, no effect on the Wnt/ß-catenin signaling pathway in DLD-1 cells (Figure 6A). On the one hand, the constitutive activation of the Wnt/ß-catenin signaling pathway and the up-regulation of its master gene target c-myc are both critical inducers of the growth potential of CRC cells including CRC stem cells [20]. On another hand, we demonstrate here, that SOX9 inhibits the growth potential of CRC cells including CRC stem cells, the activity of the Wnt/ß-catenin signaling pathway and the expression of c-myc. Thus, our data indicate that the anti-oncogenic activity of SOX9 results from a decreased expression of c-myc due to an inhibition of the activity of the oncogenic Wnt/ß-catenin signaling pathway.

### SOX9 binds with ß-catenin and induces a re-localization of ß-catenin from the chromatin to the cytosol

**Figure 7:**
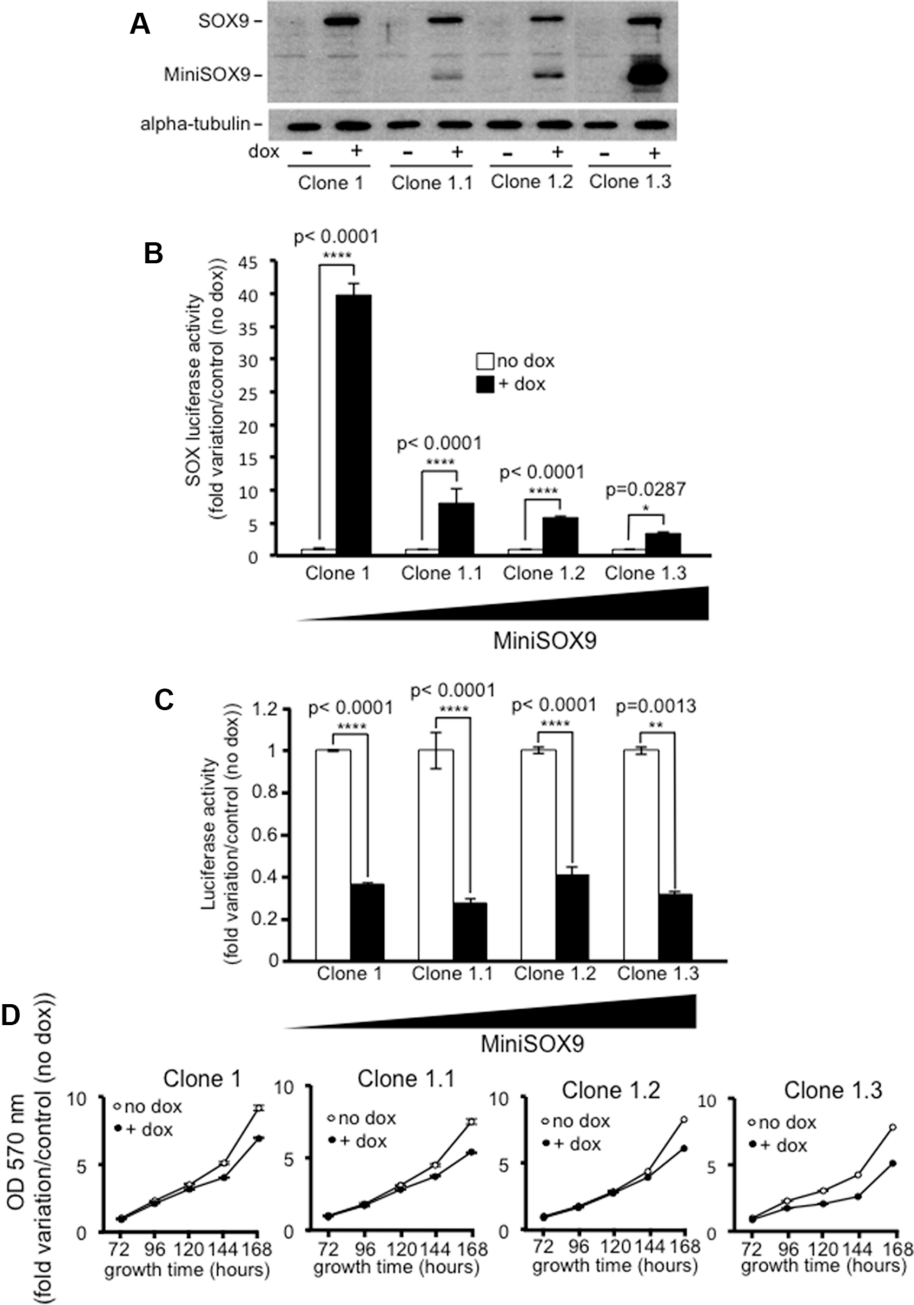
The inhibition of the Wnt/ß-catenin signaling does not require SOX9 transcription factor activity (**A**) Western blot analysis showing variable amounts of doxycycline induced MiniSOX9 expression in the S9-DLD-1 clones 1.1, 1.2 and 1.3, all isolated from the S9-DLD-1 clone 1 after infection with the MiniSOX9 lentivirus. (**B**) SOX-luciferase reporter activity (*n* = 3) in clones expressing either SOX9 alone (Clone 1) or SOX9 together with increasing amounts of MiniSOX9 (clones 1.1, 1.2 and 1.3) upon doxycycline treatment. Note that the more MiniSOX9, the more SOX9 transcriptional activity is inhibited. (**C**) Top Flash luciferase reporter assay (*n* = 3) evidencing that increasing MiniSOX9 expression levels in clones 1.1, 1.2 and 1.3 does not have any significant effect on the activity of the Wnt/ß-catenin signaling. (**D**) Crystal violet assay (*n* = 6) showing that increasing MiniSOX9 expression levels in clones 1.1, 1.2 and 1.3 does not have any significant effects on DLD-1 cells growth.

**Figure 8:**
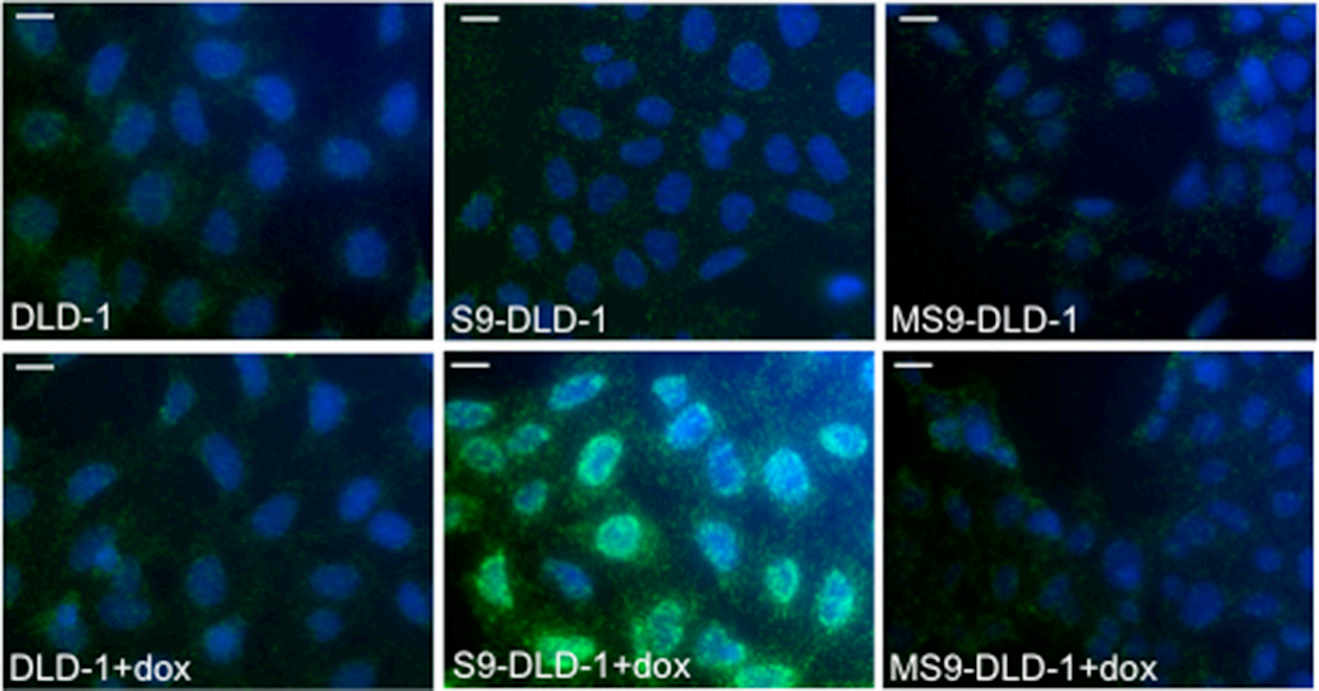
SOX9 binds with nuclear ß-catenin Duolink assay evidencing the interaction between SOX9 and ß-catenin in the nuclei of S9-DLD-1 cells (objective X40). Scale bars: 5 μm.

Previous studies reported that SOX9 transcriptional activity is not necessarily required to inhibit the activity of the Wnt/ß-catenin signaling [13, 14]. Similarly, our own data clearly demonstrate that whereas MiniSOX9 inhibits SOX9 transcriptional activity in a dose dependent manner (Figure 7A and 7B), MiniSOX9 is unable to prevent the SOX9 induced inhibition of the Top Flash dependent luciferase activity (Figure 7C) and of the DLD-1 cell growth potential (Figure 7D). We previously showed that SOX9 co-immunoprecipitates with ß-catenin [9]. Thanks to a duolink assay (Figure 8), we demonstrate here, that SOX9 physically interacts with ß-catenin in the nuclei of the doxycycline induced S9-DLD-1 cells. Cell fractionation assays (Figure S11) also further indicate a SOX9 induced decreased of chromatin-associated ß-catenin, reflecting a decrease in the ß-catenin transcriptional activity. Together, these data provide evidence that the SOX9 induced inhibition of the Wnt/ß-catenin signaling and, consequently of the cell growth, does not requires SOX9 transcriptional activity but is linked to a relocation of the nuclear ß-catenin.

### A critical dose of active SOX9 is required to maintain CRC cell proliferation

**Figure 9:**
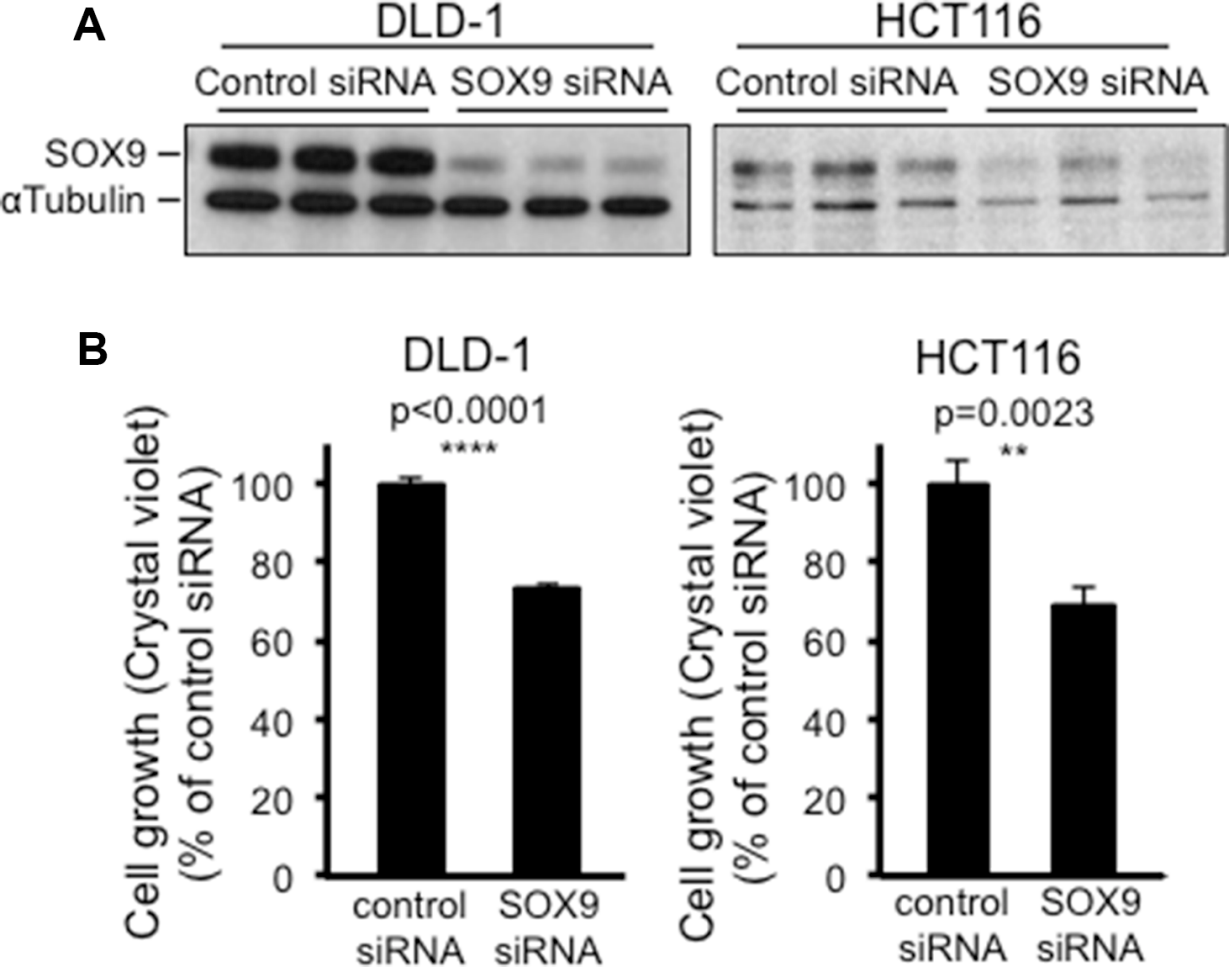
SOX9 knock down also paradoxically decreases CRC cell growth (**A**) Western blot evidencing the knock down of SOX9 due to SOX9 siRNA in both DLD-1 and HCT116 cells, and compared to a control siRNA (*n* = 3). (**B**) Crystal violet assay (*n* = 6) showing a decrease of DLD-1 and HCT116 cell growth in response to SOX9 siRNA and compared to a control siRNA.

A bimodal role for SOX9 in the intestine of healthy mice [4] has been clearly evidenced. Hence, proliferating regions associate with low levels of SOX9, whereas regions that do not proliferate can either associate with high levels of SOX9 (quiescent stem cells) or undetectable levels of SOX9 (differentiated cells). Consistent with this, our own data indicate that increasing SOX9 expression in CRC cells is also associated with a decrease of proliferation, but this might not necessarily imply that decreasing SOX9 expression is associated with an increase in CRC cell proliferation. To address this question, we used a set of four siRNAs in order to knock down SOX9 expression in both DLD-1 and HCT116 cells. As shown in Figure 9, only one of those four siRNAs efficiently decreased SOX9 expression in both DLD-1 and HCT116 cells (Figure 9A) and induced a slight decrease of cell proliferation (Figure 9B). As expected, this siRNA also decreased endogenous MiniSOX9 expression in DLD-1 and HCT116 cells (Figure S12).

**Figure 10:**
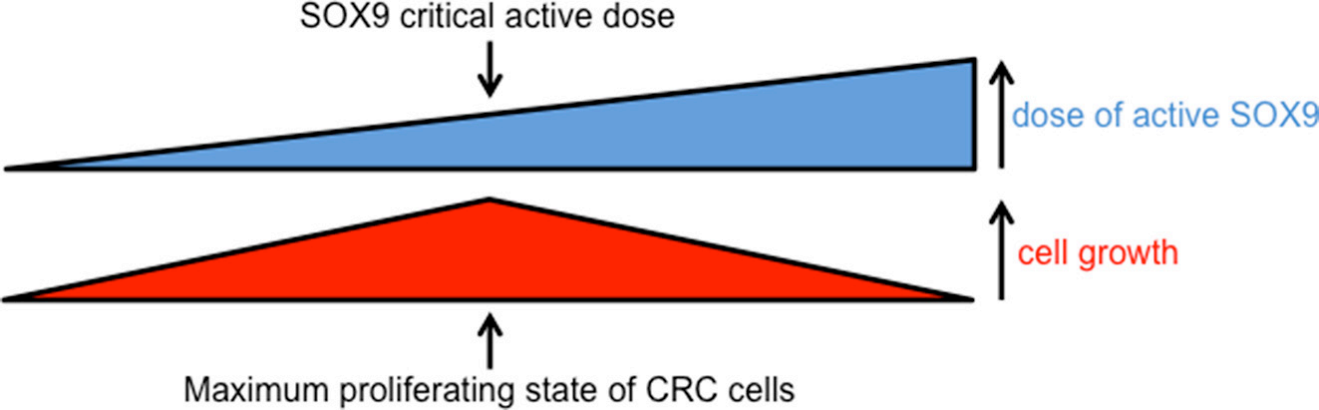
Scheme summarizing the impact of active SOX9 variations on CRC cell growth Increasing or decreasing active SOX9 levels both decrease CRC cell growth indicating that there is a critical level of SOX9 at which CRC cell growth can be maintained.

As schematized in Figure 10, these observations indicate that a critical dose of active endogenous SOX9 needs to be maintained for CRC cell proliferation while slight variations (either increases or decreases) of SOX9 levels reduce CRC cells proliferation.

## DISCUSSION

SOX9 has emerged as essential for life since inherited autosomal heterozygous mutations of the *Sox9* gene are associated with campomelic dysplasia, a severe disorder that disturbs the development of the skeleton and of the reproductive system and that is, frequently, life-threatening in the new-born period. SOX9 was also studied in the cancer context of numerous tissues [23–27] including the colon [1, 2, 5, 28]. The expression of MiniSOX9, a SOX9 splice variant that behaves as a dominant negative inhibitor of SOX9 together with the presence of inactivating mutations of SOX9 might account for the weak transcriptional activity of SOX9 in colon tumor cells [7] (Figure 1A). This also explains why despite SOX9 being a target of the Wnt/ß-catenin pathway [1] and consequently, is highly expressed in CRC compared to adjacent healthy tissues [9], SOX9 expression level was shown not to provide prognostic values and high levels of SOX9 cannot be considered as a biomarker [29]. Inversely, low levels of SOX9 at the invasive front of the primary tumor have even been shown to be an independent predictor of relapse in stage II colon cancer patients [30]. In addition, SOX9 mutations are frequent in CRC [8] and according to ICGC (http://dcc.icgc.org/web/), the *Sox9* gene is in the top 20 mutated genes with high functional impact. As shown in Figure 1A, mutations of SOX9 are present all along the sequence but it is also clear that the most impacted regions are the DNA binding domain and the two transactivations domains that are all crucial for SOX9 functional activity. Furthermore, a specific inactivation of the *Sox9* gene in the mouse intestine results in the formation of multiple microadenomas [2], thus arguing in favor of a tumor suppressor status for SOX9.

In the present study we report an additional heterozygous mutation in the human DLD-1 cell line (L142P) and evidence that this mutation results in a complete loss of SOX9 transcription factor activity as well as SOX9 capacity to inhibit the Wnt/ß-catenin pathway (Figure 1B and 1C). We postulate that this single amino acid change within the HMG domain results in an inactivating modification of SOX9 conformation and that, this mutation together with the presence of MiniSOX9 [9] is, at least in part, the cause of the poor transcriptional activity of endogenous SOX9 in DLD-1 cells (Figure 2B). Since the endogenous activity of SOX9 is negligible in DLD-1 cells, we used this model in order to investigate the phenotypic incidence of a modest increase of the dose of active SOX9 (Figures 6C and S10) by using an inducible expression system considering the relevance of comparing identical starting cells treated, or not, with doxycyline (1 μg/ml). As expected, the DLD-1 cells proliferation was strongly decreased in response to SOX9 expression (Figure 2B and 2C). In addition we also observed significant changes of the cell phenotype, such as a drastic increase of the cell size (Figure 2D and 2E), a restoration of the cell-cell contact inhibition and a decrease of the clonal expansion capacity (Figure 3A and 3B). Furthermore, we observed that SOX9 expression decreased the expansion capacity of CSC as evidenced by the lack of colonosphere formation (Figure 3C–3E). As we previously showed that SOX9 increases cell polarization [7], this may provide an explanation for DLD-1 cells growth as monolayers, cell size increase and inability to form colonospheres upon SOX9 expression.

Together, these data are undoubtedly in favor of an anti-oncogenic activity for SOX9. This was confirmed *in vivo* with subcutaneous grafts of S9-DLD-1 cells in nude mice since the tumor size was significantly reduced in response to doxycycline induced SOX9 expression (Figure S6).

The CT26 cell line [31] (grade IV carcinoma) is derived from BALB/c mice and can thus be used for syngeneic grafts in BALB/c mice which exhibit a functional immune system unlike athymic nude mice. SOX9 is not mutated in CT26 cells, but SOX9 expression is very low compared to the normal intestinal epithelium of BALB/c mice [18] and DLD-1 cells (Figure S7A). In addition, as observed for human CRC cells (Figure 2C and 2B), an induced expression of SOX9 decreased proliferation of CT26 cells (Figure 6B). Thus, CT26 cells were used to study the effect of SOX9 on tumor development in subcutaneous grafts and in a peritoneal carcinomatosis model that is the term describing widespread metastases of CRC in the peritoneal cavity. As expected, we observed a significant decrease of tumor development from grafted and doxycycline induced S9-CT26 cells (Figure 7C), indicating that SOX9 is also able to impair the metastasis potential of CRC cells. This result is further evidence that SOX9 behaves as a tumor suppressor gene although it remained unclear whether, or not, this tumor suppressor activity involved its transcription factor activity or was due to its ability to inhibit Wnt/ß-catenin activity regardless of its transcriptional activity. The present study strongly argues in favor of the second hypothesis: firstly MiniSOX9 is an inhibitor of SOX9 transcriptional activity, but is not able to prevent the SOX9 induced inhibition of both the Wnt/ß-catenin signaling pathway and the CRC cell proliferation (Figure 7). Secondly, duolink experiments showed that the SOX9 induced inhibition of Wnt/ß-catenin signaling is concomitant with SOX9 interaction with nuclear ß-catenin (Figure 8) and with ß-catenin relocation from the chromatin (Figure S11). Thirdly, inducible expression of SOX9 associates with an inhibition of the expression of c-myc, the Wnt/ß-catenin signaling prime target (Figure 6B and 6C). Since c-myc is considered as the master gene responsible for the oncogenic potential of the Wnt/ß-catenin signaling [22], we conclude that the anti-oncogenic activity of SOX9 is mainly due to an inhibition of the Wnt/ß-catenin pathway and to an inhibition of c-myc expression.

Since the question of whether SOX9 exhibits a pro- or anti-tumor activity in the intestinal epithelium is a recurring subject of controversy [32], we investigated the incidence of a decrease of endogenous SOX9 on DLD-1 and HCT116 cell growth potential and we surprisingly observed a significant decrease of cell growth (Figure 9). These experiments show that the residual quantity of active endogenous SOX9 present in DLD-1, in HCT116 and certainly more widely in CRC cells is compatible with a maximal cell proliferating state. This is in agreement with observations made with *Sox9* enhanced green fluorescent protein transgenic mice, which showed that low SOX9 expression supports a moderate proliferative capacity whereas high SOX9 expression restrains proliferation [4]. However, it is to be noticed that our experiments are apparently not in agreement with previous observations reporting an excess of proliferation in mouse intestine knocked out for the *Sox9* gene [2]. These two situations are, however, slightly different: firstly, intestinal cells of knocked out mice are not malignant tissue unlike cancer cells that exhibit a number of mutated genes; secondly SOX9 is completely absent from knocked out mice, whereas siRNA only induce a partial SOX9 knock down (Figure 9A). We do not have any evidence concerning the mechanisms involved in the cell growth decrease in response to SOX9 siRNA but, again, we can postulate that SOX9 transcription factor activity is not required in this process since MiniSOX9 has no effects on cell growth. We rather suspect interactions with other proteins; indeed SOX9 is known to physically bind to and regulate the activity of numerous transcription factors [33]. For instance we previously showed that SOX9 binds with Sp1 and, as such, decreases of PKCalpha expression [34]. Moreover, it has been recently shown that, as a co-factor of NF-Y, SOX9 is critical for the full function of NF-Y in activation of cell cycle genes [35]. This molecular mechanism is an example of a context dependent non-classical regulatory role for SOX9 on cell proliferation. Nevertheless, our findings clearly indicate that there is a crucial dose of active SOX9 at which CRC cells have a maximum proliferation rate (Figure 10). Thus, it is likely that during intestinal tumorigenesis, SOX9 expression is maintained in response to the constitutive activation of the Wnt/ß-catenin signaling pathway [1] and that the occurrence of several events (mutations, MiniSOX9 expression, decrease of expression and probably other still unknown mechanisms) durably reduce SOX9 activity. This certainly explains why SOX9 expression levels cannot be considered as a biomarker for CRC [29] and, taking into account both the MiniSOX9 expression level and the presence of SOX9 mutations, might finally be more relevant indicators of CRC severity. Nevertheless, our study gives a new insight into SOX9 function in the intestine and in CRC cancer cells.

## MATERIALS AND METHODS

### Cells

Cells doxycycline inducible for SOX9 or MiniSOX9 were established by lentiviral infection using vectors bearing the pTRIPZ inducible system (Dharmacon, Illkirch, France) and the cDNAs of interest instead of the turbo red fluorescent protein. Selection was performed with 10 ug/ml puromycin (Invivogen, Toulouse, France). Experiments were performed in the absence of puromycin, without (control) or with 1 ug/ml doxycycline (Sigma-Aldrich, Lyon, France). CT26 cells expressing luciferase were obtained with ready to use lentivirus (AMSBIO, Abingdon, UK), selection was carried out with 10 ug/ml blasticidin (Invivogen, Toulouse, France).

### Cells properties

Cell growth was evaluated from cells seeded at 50 cells per well in 96-well plates and cultured for up to 18 days, either by OD 570 nm measurements (Polarstar BMG Labtech) of cells stained by crystal violet (0.2% crystal violet; 2% ethanol) and lysed in 1% SDS or by using the Cell Titer non-radioactive cell proliferation assay (Promega, Charbonnières-les-Bains, France). Cell number, volume and diameter were measured using the Scepter^TM^ Automated Cell Counter (Millipore, Molsheim, France). Clonal capacity was evaluated by a crystal violet staining of clones grown from 1000 cells per 10 cm dishes for 11 days.

### Colonospheres

500 cells per 500 μl Serum-Free Medium (SFM: DMEM/F12 (Thermo Fisher Scientific, Saint Aubin, France), Insulin (20 μg/ml) (Sigma-Aldrich, Lyon, France), 1 % N-2 supplement (Thermo Fisher Scientific, Saint Aubin, France), EGF (20 ng/ml) (R&D systems), FGF (10 ng/ml) (R&D systems), Cyprofloxacine (2 ug/ml) (Sigma-Aldrich, Lyon, France), Gentamicine (5 ug/ml) (Thermo Fisher Scientific, Saint Aubin, France) and D-Glucose (3 mg/ml) (Sigma-Aldrich, Lyon, France) were seeded in 24 wells Corning^®^ Costar^®^ ultra-low attachment plates (Sigma-Aldrich, Lyon, France). Subcultures were made by centrifugation at 1000 rpm for 5 minutes and dissociation to single cells using Accumax (Millipore, Molsheim, France). Analyses were performed on colonospheres cultured for 11 days. Cell viability was assayed using the Cell Titer non-radioactive cell proliferation assay (Promega, Charbonnières-les-Bains, France). Colonosphere counting was performed with cultures grown from a starting concentration of 5 cells per 100 μl SFM per well in 96 plates.

### DuoLink assays

Cells were seeded on coverslips, grown for 48 h and treated with the standard immunofluorescence protocol for incubation with rabbit anti-flag and mouse anti-β-catenin primary antibodies. PLA probes, detection protocol and slide mounting were those recommended by the manufacturer (Sigma-Aldrich, Lyon, France). More information is available in supplemental materials and methods.

### Antibodies

The anti-NterSOX9/MS9 antibody was obtained using a standard rabbit immunization procedure with the synthetic peptide DTENTRPQENTFPKGC. The antibody was purified from the rabbit serum by affinity with the synthetic peptide. The anti-Cter-SOX9 antibody was previously described [5]. Anti-c-myc (9E10) and anti-α-tubulin were from home-made hybridomas. The mouse anti-flag was from Sigma-Aldrich, (Lyon, France) and the rabbit anti-β-catenin was from BD Transduction Lab (Le-Pont-de-Claix, France).

### Immunocytochemistry and Western blot

Immunocytochemistry was performed on cells plated on coverslips (50 000 cells/coverslip) and cultured for 11 days. Phalloidin was from Sigma-Aldrich (Lyon, France). A488-conjugated F(ab')2 fragment of goat anti-mouse IgG was from (Thermo Fisher Scientific, Saint Aubin, France). Preparations were mounted in Dako and observed using a Leica DM6000 microscope (Leica, Nanterre, France). Western blotting were performed using HRP conjugated anti-mouse or anti-rabbit antibodies (Millipore, Molsheim, France) and ECL (Perkin Elmer, Courtaboeuf, France). Protein expression levels were normalized relative to those of α-tubulin using the ImageJ software.

### Luciferase assays

Assays were performed as described in [1] and in supplemental materials and methods. Transfection efficiencies were normalized relative to the co-transfected phRG-TK standardization vector (Promega, Charbonnières-les-Bains, France).

### RT-QPCR analysis

Total RNA from cultured cells was isolated using the RNeasy Mini Kit (QIAGEN). One μg of total RNA was reverse transcribed using SuperScript II Reverse Transcriptase kit (Thermo Fisher Scientific, Saint Aubin, France). QRT-PCR was performed with LightCycler 480 SYBR Green I Master (Roche, Meylan, France) according to the manufacturer's instructions. Signals were detected with a Light Cycler 480 II (Roche, Meylan, France). The results were calculated using the 2^−ΔΔCt^ method, allowing for the normalization to the reference gene Mitochondrial ribosomal protein L19 (MRPL19) mRNA with the calibrator set to a value of 1 [36]. C-myc primers were from Qiagen (Courtaboeuf, France) and SOX9 primers were previously described [9].

### SiRNA

Four ON-TARGETplus SOX9 siRNAs (Dharmacon, Buckinghamshire, England) were tested but only the sequence GGAACAACCCGUCUACACA was capable of significantly decreasing SOX9 and MiniSOX9 levels. Briefly, the day before transfection, DLD-1 or HCT116 cells were seed at 4000 cells per well in 24 well plates. 40 μl of serum-free medium were incubated with 4 μl of Interferin (Polyplus Tranfection, Illkirch France) and 1 μl of ON-TARGETplus siRNA or control siRNA for 30 min at room temperature and were further added to cells in 360 μl complete medium. Seven days later, SOX9 expression and cell proliferation were evaluated, respectively, by Western blot and crystal violet staining.

### Tumorigenesis model

In agreement with the French Animal Ethics Committee (Agreement number 1152), 6 weeks old wild-type (wt) BALB/c mice (Charles River Laboratories, Saint-Germain-Nuelles, France) were injected subcutaneously with 10^6^ CT26 colon cancer cells or intraperitoneally with 2 × 10^5^ cells and treated (*n* = 8), or not (*n* = 8), with 2 mg/ml doxycycline in the drinking water. Experiments were conducted under specific pathogen-free conditions in accordance with institutional guidelines. Tumor development was monitored in real time by noninvasive bioluminescence imaging, 10 min after intraperitoneal injection of luciferin. Imaging was performed using a Camera Ivis Lumina II (PerkinElmer^®^, Courtaboeuf, France). Results are expressed in radiance (p/s/cm2/sr), which refers to the number of photons (p) per second that are leaving a square centimeter of tissue and radiating into a solid angle of 1 sr. A preliminary experiment was performed with CT26 cells expressing luciferase in order to insure that doxycycline does not reduce the tumor development (Figure S8). The experiments presented in this paper were performed with CT26 cells expressing luciferase and SOX9 upon doxycycline treatment (S9-CT26).

### Statistical analysis

Statistical analysis of *in vitro* data was performed using PRISM version 5.0 (GraphPad Software). Data are expressed as means ± SD (Student's *t*-test). Statistical analysis of *in vivo* data was performed using the Mann–Whitney *U* test. A *P*-value < 0.05 was considered significant for all comparisons.

## SUPPLEMENTARY MATERIALS




