# Supplementary material for: SOX9 is an atypical intestinal tumor suppressor controlling the oncogenic Wnt/ß-catenin signaling
Source: Oncotarget. 2016 Jul 13;7(50):82228–43. doi: 10.18632/oncotarget.10573 (PMC5347687; doi:10.18632/oncotarget.10573)
Supplement: Supplementary file 1 [file oncotarget-07-82228-s001.pdf]

SOX9 is an atypical intestinal tumor suppressor controlling the oncogenic Wnt/ß-catenin signaling

Supplementary Materials

SUPPLEMENTARY MATERIALS AND METHODS

Cell fractionation

Cell fractionation was performed as described by Wysocka *et al.* [1]. Briefly, cells were seeded as 100 000 cells per 10 cm dish, cultured for 11 days and harvested in buffer A (10 mM HEPES pH 7.9; 10 mM KCl; 1.5 mM MgCl2; 0.34 M sucrose; 10 % glycerol) with 1mM DTT, protease inhibitors (PI) and 0.1% triton X-100. After 2 min on ice, lysed cells were spun down (1300 g for 5 min) at 4°C in order to recover the supernatant S1 (cytosolic proteins). Intact nuclei (P2 pellet) were washed with buffer A, spun down as above, re-suspended in buffer B (3 mM EDTA; 0,2 mM EGTA) with 1 mM DTT and PI, kept on ice for 30 min and spun down (1700 g for 5 min) at 4°C in order to recover the supernatant S2 (nuclear soluble proteins). The P3 pellet (chromatin fraction) was washed with buffer B, spun down as above and re-suspended in buffer B. Equivalent volumes of S1, S2 and P3 fractions diluted in Laemli were analyzed by western blot.

Luciferase assays

The "SOX-luciferase" reporter construct contains seven copies of the AACAAAG sox-binding sequence, inserted upstream of a minimal herpes simplex thymidine kinase promoter; the control "SAC-luciferase" exhibits seven copies of the CCGCGGT sequence (generous gift from Prof. H. Clevers).

The TOPFlash luciferase reporter of ß-catenin-mediated transcriptional contains 4 copies of TCF/LEF binding sites (AGATCAAAGG) inserted upstream of a minimal herpes simplex thymidine kinase promoter (generous gift from Prof. H. Clevers).

DuoLink assay

The Duolink assay allows detection, visualization and quantification of protein-protein interactions in fixed cells. The targeted proteins are detected by using primary antibodies from different species and coupled to oligonucleotide probes suitable for PLA (Proximity Ligation Assay). The signal can be generated only when the PLA probes are in close proximity, thereby allowing ligation and amplification using fluorescent nucleotides. The resulting fluorescent dots reflect the oligonucleotides ligation events and thus the interactions between proteins targeted with antibodies.

REFERENCES

1. Wysocka J, Reilly PT, Herr W. Loss of HCF-1-chromatin association precedes temperature-induced growth arrest of tsBN67 cells. Molecular and cellular biology. 2001; 21:3820–3829.

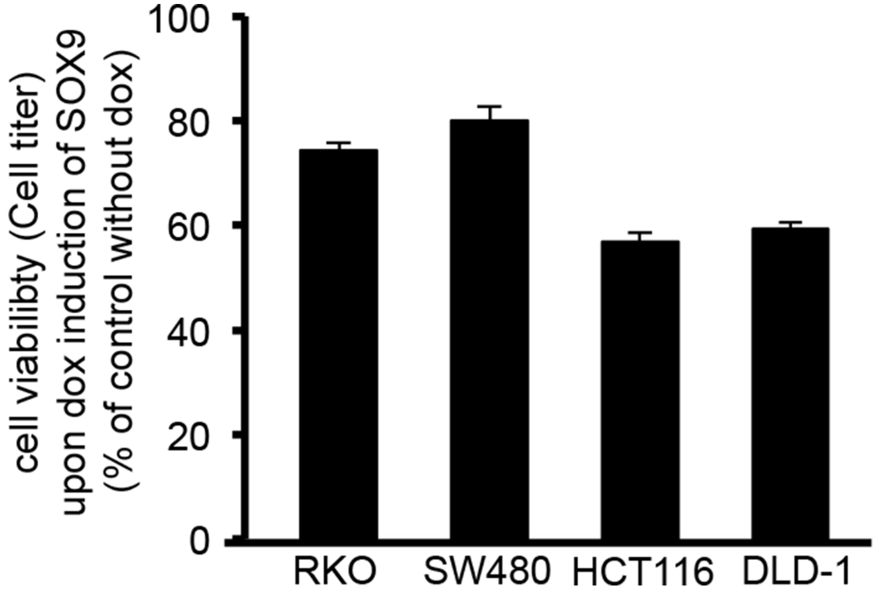

Supplementary Figure S1: Cell titer assay (*n* = 6) showing a decrease of RKO, SW480, HCT116 and DLD1 cell viability in response to doxycycline-induced SOX9 expression.

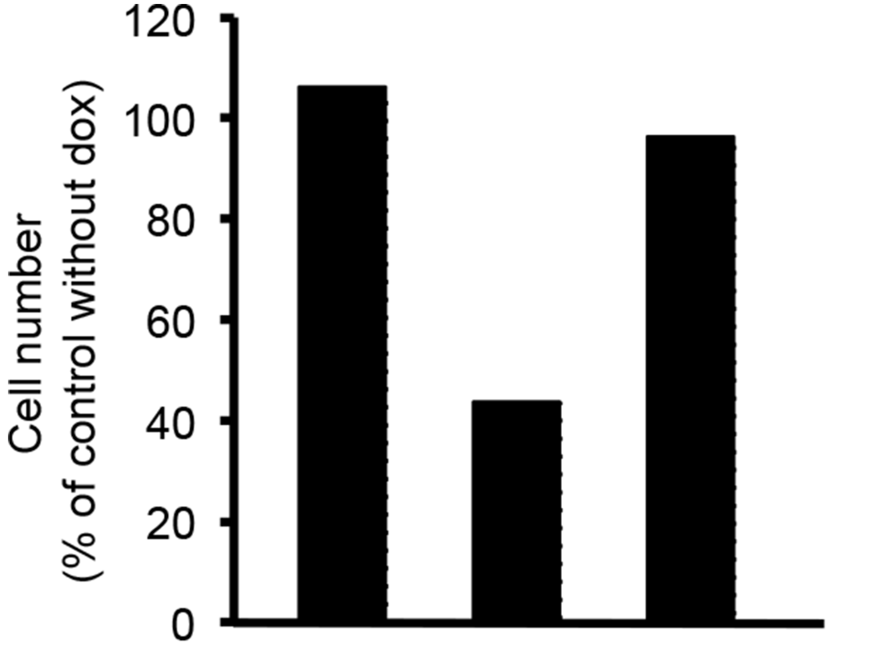

Supplementary Figure S2: A representative experiment of normalized scepter data illustrating the decrease of the cell number in response to doxycycline induced SOX9 expression while doxycycline induced MiniSOX9 expression has no significant effects.

Mutations	RKO	SW480	HCT116	DLD-1
KRAS	-	+	+	+
BRAF	+	-	-	-
APC	-	+	-	+
ß-catenin	-	-	+	-
SOX9	+*	-	-	+

* p.Q195fs*58 (insersion-frameshift)

Supplementary Figure S3: Census of mutated KRAS, BRAF, APC, ß-catenin and SOX9 in RKO, SW480, HCT116 and DLD-1 cell lines according to http://cancer.sanger.ac.uk/cancergenome/projects/cell_lines/.

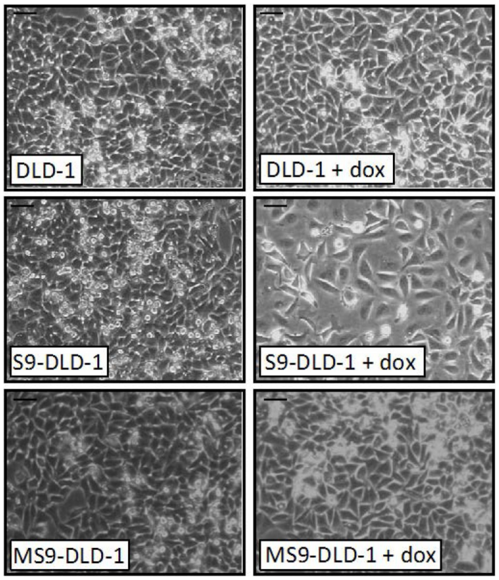

Supplementary Figure S4: Phase contrast images of DLD-1, S9-DLD-1 and MS9-DLD-1 cells cultured for 11 days in either the absence or presence of doxycycline (objective X20).

	DLD-1		S9-DLD-1		MS9-DLD-1	
Dox	-	+	-	+	-	+
Cell number (per ml)	565000	600800	682400	299300	544200	524500
Cell volume (pl)	2.56	2.551	1.841	3.78	2.503	2.69
Cell diameter (µm)	17.18	16.95	15.2	18.04	16.84	17.25

Supplementary Figure S5: Raw data of Scepter measurements performed with DLD-1, S9-DLD-1 and MS9-DLD-1 cells cultured for 11 days in either the absence or presence of doxycycline.

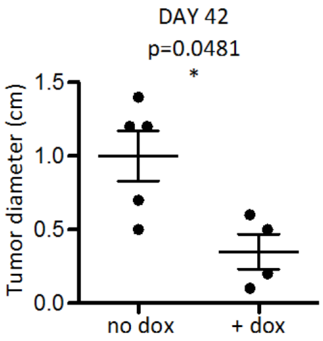

Supplementary Figure S6: Measurement of the size of the tumors grown from subcutaneous grafts of S9-DLD-1 cells in nude mice.

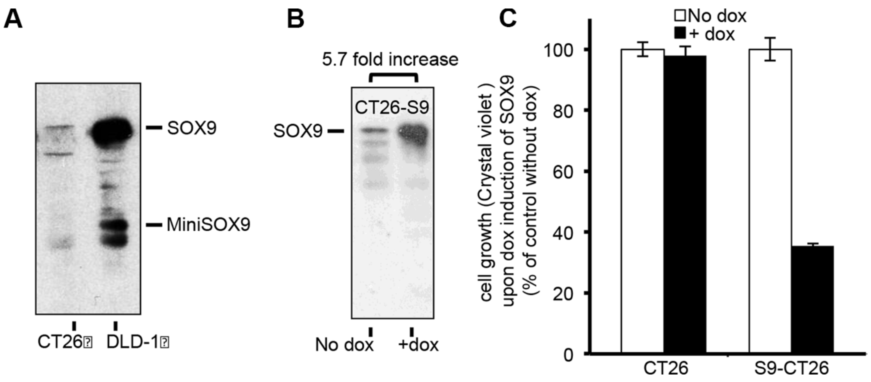

Supplementary Figure S7: (**A**) Comparison of SOX9 expression levels in CT26 and DLD-1 cells (40 000 cells) (**B**) Western blot analysis of the doxycycline induced level of SOX9 expression for the CT26-S9 clone (40 000 cells) selected for syngeneic grafts in BALB/c mice. The increase of SOX9 expression was visualized with the anti-Cter-SOX9 antibody and estimated thanks to the ImageJ software. (**C**) Crystal violet assay (n = 6) evidencing SOX9 induced inhibition of CT26 cells growth upon doxycycline induction.

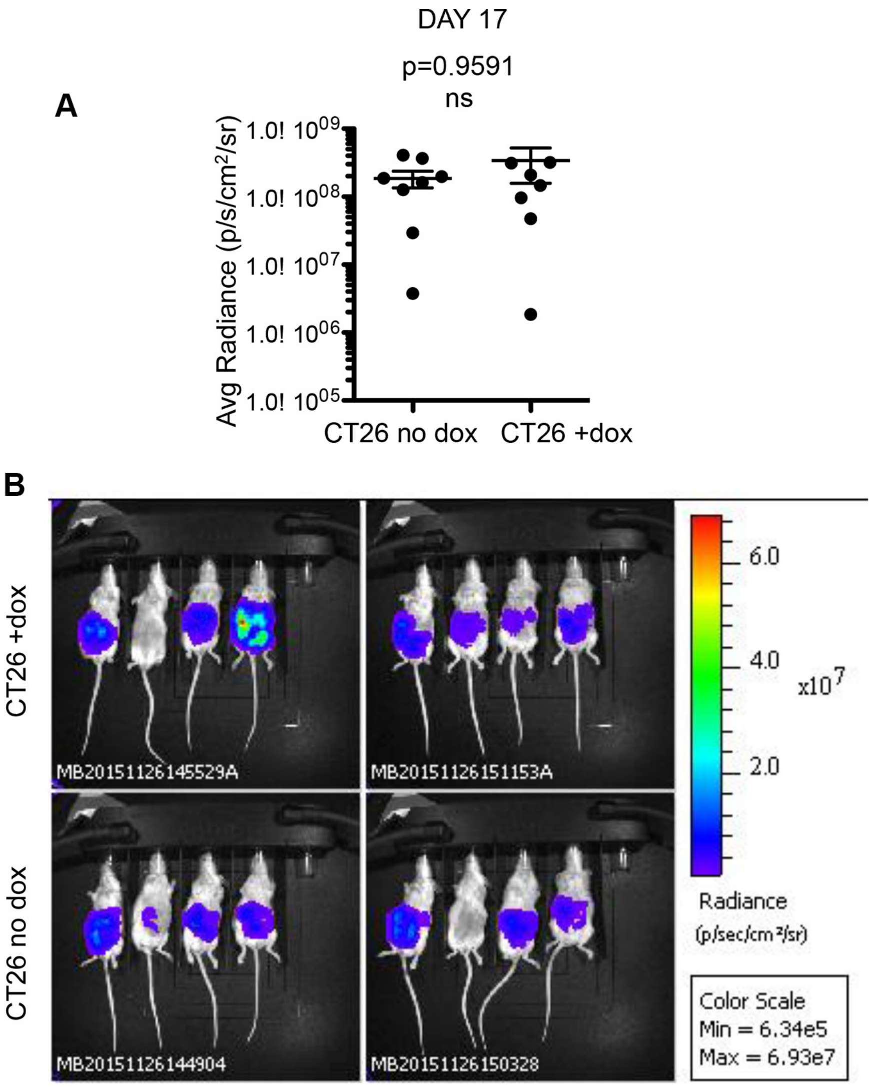

Supplementary Figure S8: (**A**) Quantification of the bioluminescence signal (average radiance (p/s/cm2/sr)) related to the development of tumors 17 days after intraperitoneal transplantation of CT26 cells, upon doxycycline treatment (n = 8) or not (n = 8). (**B**) Bioluminescence imaging 17 days after intraperitoneal transplantation of S9-CT26 cells.

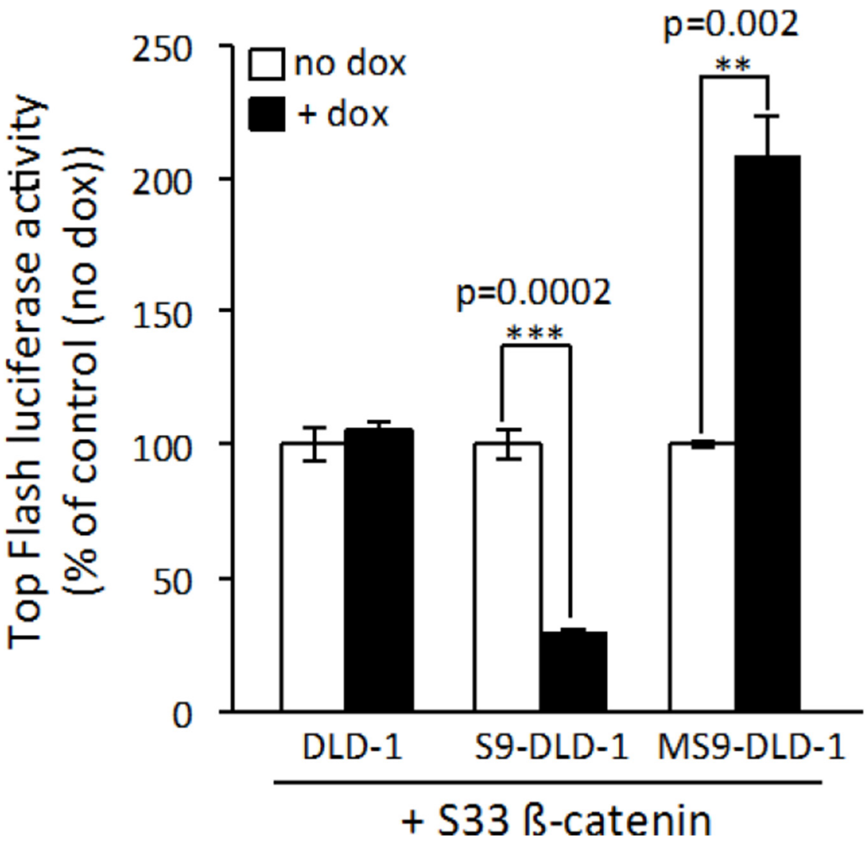

Supplementary Figure S9: Top Flash luciferase reporter assay (*n* = 3) evidencing the decrease of the activity of the Wnt/ß-catenin signaling upon doxycycline induced SOX9 and the increase of activity upon induced MiniSOX9 expression when co-transfecting the stabilized ^{33}S ß-catenin.

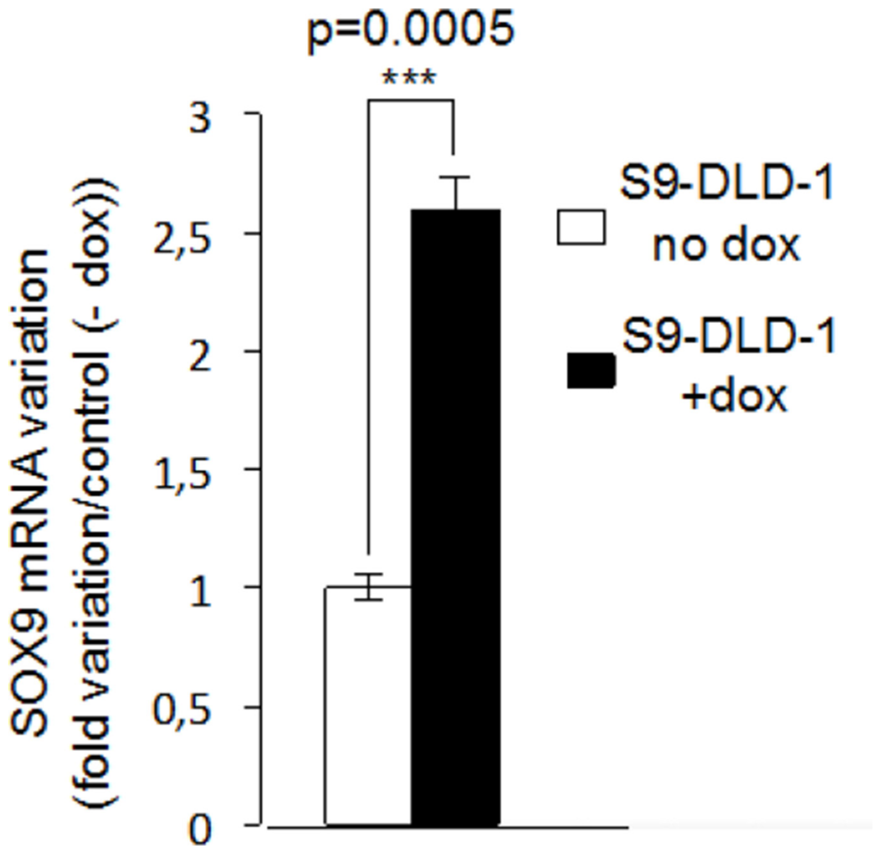

Supplementary Figure S10: Quantitative RT-PCR analysis attesting the increase of SOX9 mRNA in response to doxycycline induced SOX9 expression (*n* = 3).

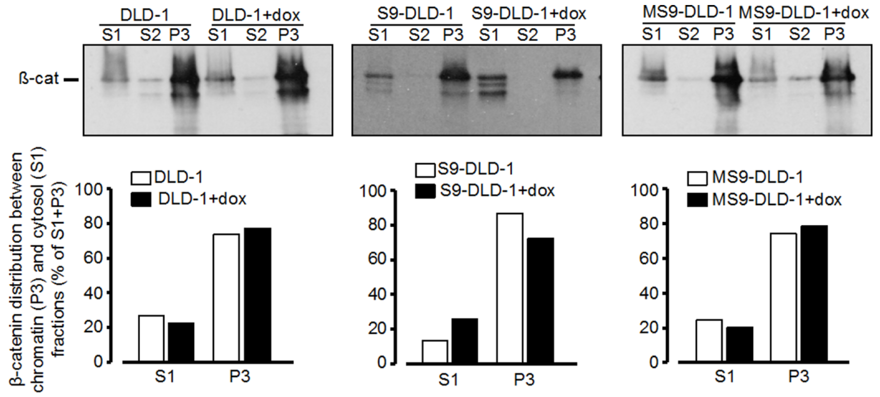

Supplementary Figure S11: A representative Western blot experiment showing ß-catenin re-localization from the chromatin (P3) to the cytosolic fraction (S1) in response to doxycycline induced SOX9 expression (upper panel). Note the absence of significant changes of the ß-catenin levels associated with P3 and S1 for DLD-1 and MS9-DLD-1 cells or with S2 (nucleosolic fraction) for DLD-1, S9-DLD-1 and MS9-DLD-1 cells upon doxycycline induction. Lower panels show a quantification of the ß-catenin signals in S1 and P3 fractions by using the ImageJ software (lower panel).

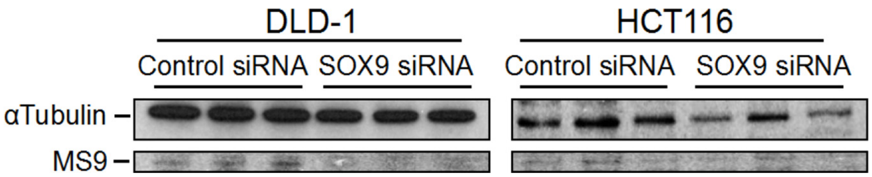

Supplementary Figure S12: Western blot evidencing the knockdown of MiniSOX9 due to SOX9 siRNA in both DLD-1 and HCT116 cells, and compared to a control siRNA (*n* = 3).

Supplementary Table S1: The potential impact of mutations on SOX9 activity. See Supplementary_ Table_S1
