# Supplementary material for: SOX9 is an atypical intestinal tumor suppressor controlling the oncogenic Wnt/ß-catenin signaling
Source: Oncotarget. 2016 Jul 13;7(50):82228–43. doi: 10.18632/oncotarget.10573 (PMC5347687; doi:10.18632/oncotarget.10573)
Supplement: Supplementary file 2 [file oncotarget-07-82228-s002.doc]

| **Tissue** | **AA Mutation** | **CDS Mutation** | | **Impact on protein** | | **Probable impact on activity** |
| --- | --- | --- | --- | --- | --- | --- |
| Caecum | p.N132S | c.395A>G | | Single aminoacid subtstitution HMG | | unknown |
| Caecum | p.Q339* | c.1015C>T | | truncation | | dominant negative |
| Caecum | p.D244fs*7 | c.729_736CGACGTGC>A | | truncation | | dominant negative |
| Caecum | p.W115* | c.344G>A | | truncation | | loss |
| Caecum | p.I435fs*35 | c.1300delC | | truncation | | dominant negative |
| Caecum | p.D441fs*>70 | c.1320_1321insG | | truncation | | dominant negative |
| Caecum | p.F423fs*47 | c.1267delT | | truncation | | dominant negative |
| Caecum | p.A31V | c.92C>T | | Single aminoacid subtstitution | | unknown |
| Caecum | p.E63fs*189 | c.183_184insA | | truncation | | loss |
| Caecum | p.? | c.432-13_452del34 | |  | | loss |
| Caecum | p.R394* | c.1180C>T | | truncation | | dominant negative |
| Caecum | p.E293fs*4 | c.871_872insTCAA | | truncation | | dominant negative |
| Caecum | p.S214fs*1 | c.640_652del13 | | truncation | | dominant negative |
| Caecum | p.S408fs*64 | c.1215_1216insCACTA | | truncation | | dominant negative |
| Caecum | p.H406fs*>105 | c.1216_1217insA | | truncation | | dominant negative |
| Caecum | p.R94H | c.281G>A | | Single aminoacid subtstitution | | unknown |
| Colon | p.F270L | c.810C>A | | Single aminoacid subtstitution | | unknown |
| Colon | p.S27S | c.81C>T | | synonym | | active |
| Colon | p.N110S | c.329A>G | | Single aminoacid subtstitution HMG | | unknown |
| Colon | p.E255fs*40 | c.761_762delGA | | truncation | | dominant negative |
| Colon | p.E388fs*1 | c.1155_1197del43 | | truncation | | dominant negative |
| Colon | p.N470D | c.1408A>G | | Single aminoacid subtstitution | | unknown |
| Colon | p.P307fs*77 | c.915_916insGT | | truncation | | dominant negative |
| Colon | p.D290fs*6 | c.866_867insC | | truncation | | dominant negative |
| Colon | p.Y174C | c.521A>G | | Single aminoacid subtstitution HMG | | unknown |
| Colon | p.P176S | c.526C>T | | Single aminoacid subtstitution HMG | | unknown |
| Colon | p.Q230fs*23 | c.684delG | | truncation | | dominant negative |
| Colon | p.R152delR | c.453_455delGCG | | Single aminoacid deletion HMG | | unknown |
| Colon | p.P258fs*23 | c.764_765insGGGGC | | truncation | | dominant negative |
| Colon | p.R257fs*39 | c.764_765insG | | truncation | | dominant negative |
| Colon | p.H413fs*57 | c.1238_1241ACTC>TGA | | truncation | | dominant negative |
| Colon | p.Y172H | c.514T>C | | Single aminoacid subtstitution HMG | | unknown |
| Colon | p.R264fs*32 | c.782_783insG | | truncation | | dominant negative |
| Colon | p.Y440fs*>71 | c.1316_1317insG | | truncation | | dominant negative |
| Colon | p.W115fs*136 | c.340_341delGT | | truncation HMG | | loss |
| Colon | p.? | c.686-1G>A | | ? | | ? |
| Colon | p.V306fs*>205 | c.911_912insG | | truncation | | dominant negative |
| Colon | p.S448fs*1 | c.1343_1344delCC | | truncation | | dominant negative |
| Colon | p.V105G | c.314T>G | | Single aminoacid subtstitution HMG | | unknown |
| Colon | p.S23fs*38 | c.62delC | | truncation before HMG | | loss |
| Colon | p.T465T | c.1395C>G | | synonym | | active |
| Colon | p.K173_Y174ins* | c.517_518insAAT | | Single aminoacid insersion HMG | | unknown |
| Colon | p.Q417* | c.1249C>T | | truncation | | dominant negative |
| Colon | p.H396fs*8 | c.1183_1184insCG | | truncation | | dominant negative |
| Colon | p.Q164fs*20 | c.486_487insGT | | truncation HMG | | loss |
| Colon | p.Q401* | c.1201C>T | | truncation | | dominant negative |
| Colon | p.D274fs*6 | c.816_817insGT | | truncation | | dominant negative |
| Colon | p.A116V | c.347C>T | | Single aminoacid subtstitution HMG | | unknown |
| Colon | p.A189V | c.566C>T | | Single aminoacid subtstitution | | unknown |
| Colon | p.Q265* | c.793C>T | | truncation | | dominant negative |
| Colon | p.W335* | c.1005G>A | | truncation | | dominant negative |
| Colon | p.T478fs*>33 | c.1430_1431insC | | truncation | | dominant negative |
| Colon | p.Y172fs*80 | c.512_513insT | | truncation HMG | | loss |
| Colon | p.P237fs*14 | c.707_708delCC | | truncation | | dominant negative |
| Colon | p.T243fs*10 | c.719delC | | truncation | | dominant negative |
| Colon | p.A308S | c.922G>T | | Single aminoacid subtstitution | | unknown |
| Colon | p.E148* | c.442G>T | | truncation HMG | | loss |
| Colon | p.G256fs*24 | c.760_761insGA | | truncation | | dominant negative |
| Colon | p.Y420C | c.1259A>G | | Single aminoacid subtstitution | | unknown |
| Colon | p.G390fs*14 | c.1164_1165insCC | | truncation | | dominant negative |
| Colon | p.G233fs*20 | c.696delG | | truncation | | dominant negative |
| Colon | p.Q230* | c.688C>T | | truncation | | dominant negative |
| Colon | p.P415fs*56 | c.1243_1244insGC | | truncation | | dominant negative |
| Colon | p.Q416fs*54 | c.1243delC | | truncation | | dominant negative |
| Colon | p.Q458fs*>53 | c.1370_1371insC | | truncation | | dominant negative |
| Colon | p.R254* | c.760C>T | | truncation | | dominant negative |
| Colon | p.P346fs*37 | c.1033delC | | truncation | | dominant negative |
| Colon | p.K249fs*5 | c.742_743insGC | | truncation | | dominant negative |
| Colon | p.S414* | c.1241C>A | | truncation | | dominant negative |
| Colon | p.E409fs*61 | c.1225delG | | truncation | | dominant negative |
| Colon | p.Q412delQ | c.1226_1228delAGC | | Single aminoacid deletion | | unknown |
| Colon | p.R254Q | c.761G>A | | Single aminoacid subtstitution | | unknown |
| Colon | p.A158V | c.473C>T | | Single aminoacid subtstitution HMG | | unknown |
| Colon | p.V306fs*78 | c.911_912insGG | | truncation | | dominant negative |
| Colon | p.Y428C | c.1283A>G | | Single aminoacid subtstitution | | unknown |
| Colon | p.A118T | c.352G>A | | Single aminoacid subtstitution HMG | | unknown |
| Colon | p.F112L | c.336C>A | | Single aminoacid subtstitution HMG | | unknown |
| Colon | p.W86* | c.258G>A | | truncation HMG | | loss |
| Colon | p.K166delK | c.496_498delAAG | | Single aminoacid deletion HMG | | unknown |
| Colon | p.Q439fs*31 | c.1314delA | | truncation | | dominant negative |
| Colon | p.K68E | c.202A>G | | Single aminoacid subtstitution | | unknown |
| Colon | p.P258fs*22 | c.767_768insGC | | truncation | | dominant negative |
| Colon | p.R120C | c.358C>T | | Single aminoacid subtstitution HMG | | unknown |
| Colon | p.S181P | c.541T>C | | Single aminoacid subtstitution HMG | | unknown |
| Colon | p.P367fs*16 | c.1096delC | | truncation | | dominant negative |
| Colon | p.Q246* | c.736C>T | | truncation | | dominant negative |
| Colon | p.K61fs*191 | c.178_179insT | | truncation HMG | | loss |
| Colon | p.K141E | c.421A>G | | Single aminoacid subtstitution HMG | | unknown |
| Colon | p.Q175P | c.524A>C | | Single aminoacid subtstitution HMG | | unknown |
| Colon | p.E400fs*>111 | c.1196_1197insG | | truncation | | dominant negative |
| Colon | p.V77F | c.229G>T | | Single aminoacid subtstitution | | unknown |
| Rectum | p.V486fs*>25 | c.1452_1453insG | | truncation | | dominant negative |
| Rectum | p.G263fs*16 | c.783delG | | truncation | | dominant negative |
| Rectum | p.V77L | c.229G>C | | Single aminoacid subtstitution | | unknown |
| Rectum | p.Y428fs*>75 | c.1279_1301del23 | | truncation | | dominant negative |
| Rectum | p.R257fs*23 | c.764_765insGG | | truncation | | dominant negative |
| Rectum | p.N201fs*51 | c.595_596insC | | truncation | | dominant negative |
| Rectum | p.I480fs*>31 | c.1429_1430insAC | | truncation | | dominant negative |
| Rectum | p.Q412* | c.1234C>T | | truncation | | dominant negative |
| Rectum | p.R271fs*25 | c.809_810insC | | truncation | | dominant negative |
| Rectum | p.Q369* | c.1105C>T | | truncation | | dominant negative |
| Rectum | p.Q411* | c.1231C>T | | truncation | | dominant negative |
| Rectum | p.P235fs*19 | c.698_699insCC | | truncation | | dominant negative |
| Rectum | p.T236fs*24 | c.686_687ins20 | | truncation | | dominant negative |
| Rectum | p.R394fs*>117 | c.1179_1180insC | | truncation | | dominant negative |
| Rectum | p.P434fs*38 | c.1291_1292insCCTAC | | truncation | | dominant negative |
| Colon | p.R120L | c.359G>T | | Single aminoacid subtstitution HMG | | unknown |
| Colon | p.V291fs*92 | c.872delT | | truncation | | dominant negative |
| Colon | p.L252fs*37 | c.753_1035del283 | | truncation | | dominant negative |
| Colon | p.P377fs*3 | c.1130_1140>G | | truncation | | dominant negative |
| Colon | p.L146fs*37 | c.437delT | | truncation HMG | | loss |
| Colon | p.A379fs*5 | c.1133_1134insGG | | truncation | | dominant negative |
| Colon | p.Q208* | c.622C>T | | truncation | | dominant negative |
| Colon | p.L135F | c.403C>T | | Single aminoacid subtstitution HMG | | unknown |
| Colon | p.Y451fs*>61 | c.1347_1348insTCCT | | truncation | | dominant negative |
| Colon | p.V306fs*77 | c.912delG | | truncation | | dominant negative |
| Colon | p.T25fs*37 | c.68_69insCC | | truncation before HMG | | loss |
| Colon | p.E409* | c.1225G>T | | truncation | | dominant negative |
| NS | p.Y451fs*19 | c.1349delC | | truncation | | dominant negative |
| NS | p.I198fs*22 | c.591_592insTC | | truncation | | dominant negative |
| NS | p.L259fs*20 | c.771delC | | truncation | | dominant negative |
| NS | p.? | c.685+1_685+2delGT | | ? | | unknown |
| NS | p.K68K | c.204G>A | | synonym | | active |
| NS | p.P415fs*56 | c.1240_1241insCG | | truncation | | dominant negative |
| NS | p.T11A | c.31A>G | | Single aminoacid subtstitution | | unknown |
| NS | p.Q401fs*3 | c.1199_1200insGC | | truncation | | dominant negative |
| NS | p.M476fs*>35 | c.1421_1422insC | | truncation | | dominant negative |
| NS | p.Q195fs*58 | c.579_580insGAGC | | truncation | | dominant negative |
| NS | p.V486A | c.1457T>C | | Single aminoacid subtstitution | | unknown |
| NS | p.A111V | c.332C>T | | Single aminoacid subtstitution HMG | | unknown |
| NS | p.P267fs*12 | c.796delC | | truncation | | dominant negative |
| NS | p.R94H | c.1277_1278insGA | | truncation | | dominant negative |
| red: probable loss of activity due to truncation in the HMG (DNA binding site) or before the HMG domain. | | | | |  | |
| Yellow: Truncations dowstream the HMG domain probably resulting in loss of activity and in a dominant negative protein, like for MiniSOX9. | | | | | |  |
| 91 probable loss of activity with 80 likely dominant negative variants | | |  | | | |
